# The diagnostic accuracy of soft tissue oedema measurements: a systematic review and best-evidence synthesis

**DOI:** 10.1007/s00520-026-10373-y

**Published:** 2026-03-05

**Authors:** Margje B. Buitenhuis, Elise M. Gane, Janine T. Hidding, Judith D. de Rooij, Wichor M. Bramer, Remco de Bree, Caroline M. Speksnijder

**Affiliations:** 1 Department of Head and Neck Surgical Oncology, University Medical Center Utrecht, Utrecht University, Utrecht, The Netherlands; 2 Department of Oral and Maxillofacial Surgery, University Medical Center Utrecht, Utrecht University, G05.122, P.O. Box 85.500, Utrecht, 3508 GA The Netherlands; 3 Physiotherapy Department, Princess Alexandra Hospital, Brisbane, Australia; 4 Centre for Functioning and Health Research, Metro South Health, Brisbane, Australia; 5 School of Health and Rehabilitation Sciences, The University of Queensland, Brisbane, Australia; 6 Physiotherapy Department, Avans University of Applied Sciences, Breda, The Netherlands; 7 Department of Orthopaedics, Physiotherapy Unit, Erasmus MC, Erasmus University Medical Center, Rotterdam, The Netherlands; 8 Medical Library, Erasmus MC, Erasmus University Medical Center, Rotterdam, The Netherlands

**Keywords:** Diagnosis, Oedema, Lymphoedema, Sensitivity, Specificity

## Abstract

**Purpose:**

Effective lymphoedema management relies on early detection and treatment during its reversible phase, underlining the importance of accurate measurement tools. This systematic review aims to identify measurement instruments for quantitatively diagnosing lymphoedema and their diagnostic accuracy.

**Methods:**

Literature was systematically searched on the diagnostic accuracy of instruments for assessing soft tissue oedema across body parts in adults. Inclusion criteria encompassed studies establishing diagnostic accuracy (sensitivity and/or specificity) of instruments for quantifying oedema through volume changes, tissue characteristics, or lymphatic system function. Searches included Embase, Medline, Web of Science, and CINAHL databases from inception to March 18, 2024. Methodological quality was assessed using the COSMIN checklist for criterion validity and QUADAS-2. Diagnostic value was evaluated through the Youden index, and the level of evidence was established using a new best-evidence synthesis approach.

**Results:**

A total of 44 studies were included, identifying 14 index measurement instruments. The most frequently studied instruments were tape measurements, ultrasound, and multi-frequency bio-impedance analysis (MF-BIA). Instruments with very high diagnostic value (Youden index ≥ 0.90) included MF-BIA, perometry, and MRI. However, the quality of evidence supporting these instruments was lacking. Nine different instruments served as references, with tape measurements, consensus criteria, and water volumetry being the most applied.

**Conclusion:**

This review underscores the complexity of accurately diagnosing lymphoedema, with no single instrument emerging as a definitive gold standard. Clinicians must weigh the available evidence and consider the clinical context, such as early detection, when selecting measurement instruments for diagnosing lymphoedema.

**Supplementary Information:**

The online version contains supplementary material available at 10.1007/s00520-026-10373-y.

## Introduction

Lymphoedema is a significant global health issue, affecting approximately 200 million people worldwide [1]. This condition is characterized by lymphatic fluid accumulation and occurs as either primary, due to genetic mutations, or secondary, arising from systemic disease, trauma, or interventions, especially cancer-related surgeries and radiotherapy [2–5].

The International Society of Lymphoedema (ISL) stages lymphoedema from subclinical (stage 0) to hard, fibrotic oedema (stage 3) [2]. Early symptoms are often subjective, like tightness or heaviness, progressing to visible swelling and tissue fibrosis [2]. Chronic lymphoedema can cause significant physical discomfort, including pain, swelling, heaviness, and reduced mobility of the affected area, and is associated with complications such as recurrent skin infections, skin ulcers, or, in the worst case, angiosarcoma [5]. Lymphoedema has major psychological and social impacts on the quality of life of patients [6]. Moreover, the condition is a major burden on the health care system because it is a chronic and progressive condition that often requires lifelong treatment [5].

Lymphoedema treatment is commonly a multi-modal approach of manual lymphatic drainage, compression, exercises, and skincare [7, 8]. Several adjunct treatment options have also been trialled, such as pneumatic compression, pharmaceuticals, laser therapy, and microvascular surgery [7]. Evidence suggests that early treatment of lymphoedema is both effective and cost-saving [4]. To commence early treatment of lymphoedema, early detection is crucial, underscoring the need for measurement instruments with good diagnostic capacity.

Lymphoedema can be quantified in multiple methods, including volume, tissue characteristics, and lymphatic system function, depending on its stage [2, 5]. However, no universally accepted gold standard for assessment exists, and multiple instruments with unique advantages and drawbacks are applied in research and clinical settings [9].

To our knowledge, only one systematic review has evaluated the diagnostic accuracy of lymphoedema measurement instruments [9]. This review highlighted limited and sometimes conflicting results for measuring upper extremity lymphoedema and recommended water volumetry as the reference test due to its superior reliability. Although various instruments were assessed, only one study used water volumetry as the reference, evaluating tape measurement with sensitivities from 0.05 to 0.90 and specificities from 0.69 to 1.00. Notably, advanced imaging modalities like computed tomography (CT), magnetic resonance imaging (MRI), and ultrasound (US) were excluded, though these could provide valuable information and could be applied in specific cases during regular clinical follow-up.

Given advancements in lymphoedema research since this review from 2015, a reassessment of measurement instruments and their properties is relevant and timely. This systematic review aims to identify instruments for quantitatively diagnosing soft-tissue oedema, including lymphoedema, across any body part and their diagnostic accuracy. We expect the results to assist clinicians in selecting appropriate instruments for their patients and support researchers in improving early diagnostic methods.

## Methods

### Protocol and registration

This review follows the Preferred Reporting Items for Systematic Reviews and Meta-Analyses (PRISMA) guidelines [10] and has been registered in PROSPERO (registration number: CRD42023474209). Given the extensive initial search, this review addresses the diagnostic accuracy of measurement instruments for soft tissue oedema, with other clinimetric properties covered in separate reviews (in preparation).

### Eligibility criteria

Studies were included when describing measurement instruments that quantified soft tissue oedema. Although the primary focus was lymphoedema, studies assessing other types of soft tissue swelling, such as postoperative swelling that could mimic early-stage lymphoedema, were also included. Studies were excluded when describing measurements of temporary oedema in healthy subjects (e.g. individuals with standing jobs or those taking long flights), although cohort studies with patients and healthy subjects were included. Studies reporting on soft tissue oedema in skin conditions (e.g. ulcers, burn wounds, or psoriasis) or vascular oedema were excluded. Only prospective studies involving adults (≥ 16 years of age) were included; studies on paediatric cohorts were excluded. Instruments had to measure body part or tissue volume, tissue characteristics, or lymphatic system function. Clinical assessment of one of these aspects by a specialist was also considered relevant. Patient-reported outcomes were excluded. Studies on measurement instruments not aiming to quantify soft tissue oedema or those quantifying the quality of the venous system were excluded. Studies that reported both objective measures and patient-reported measures of lymphoedema were included, with only objective data extracted.

Studies had to report on the diagnostic accuracy with sensitivity or specificity. For cohort studies, data had to be presented separately for patients, patients at risk, and healthy subjects. Data was excluded when patients (at risk) and healthy subjects had a difference in the mean age of more than 10 years to minimize bias as aging leads to alteration of elastic properties of the skin [11]. Meta-analyses, systematic reviews, conference papers, case reports, and studies without accessible full text were excluded.

### Information sources and search

Electronic databases Embase.com, Medline via Ovid, Web of Science Core Collection, and CINAHL via EBSCO were systematically searched from inception until March 18, 2024 (date last searched). The search strategy included terms related to the construct (e.g. lymphoedema, swelling) combined with a methodological search filter for finding studies on measurement properties [12]. Full search strategies are provided in Supplementary information 1.

### Study selection

Two reviewers (MBB and JTH) independently assessed titles and abstracts of the studies retrieved by the literature search. Full-text articles were then retrieved and divided into two sets for examination. Each set was independently reviewed by two reviewers (MBB and EMG for set A; JTH and JDR for set B) to compile the final list of eligible studies. Disagreements were resolved by a third reviewer (CMS).

### Data collection process

Two reviewers (MBB and EMG) independently extracted data from the included articles into a custom-built template in Microsoft Word. Extracted data included the first author, year of publication, index measurement instrument, reference measurement instrument, study population (age, gender, previous diagnosis, swelling type, and swelling stage), and diagnostic accuracy (sensitivity and specificity). Consensus was achieved through discussion in cases of disagreement.

### Quality assessment

The methodological quality of included studies was independently assessed by two reviewers (MBB and EMG) using the Quality Assessment of Diagnostic Accuracy Studies tool (QUADAS-2), which evaluates patient selection, index test, reference standard, and flow and timing [13]. Each domain contributes to the assessment of risk of bias, with the first three domains also assessing applicability. In addition, criterion validity was specifically evaluated using the COnsensus-based Standards for the selection of health Measurement Instruments (COSMIN) checklist Box 8 [14]. The assessment protocol is detailed in Supplementary information 2. Disagreement was resolved by a third reviewer (CMS).

### Data synthesis

Pooling of the studies was not possible due to the diversity in instruments and parameters. Therefore, a best-evidence synthesis for diagnostic accuracy has been established and conducted. Index measurement instruments are categorized into five levels of evidence as presented in Table 1.

**Table 1:** Best-evidence synthesis for diagnostic accuracy

Level of evidence	Criteria
Strong evidence	Provided by at least three studies with very good quality, defined as ≥ 3 good quality-scores (+) for QUADAS-2 Risk of bias, **and** 3 good-quality scores (+) for QUADAS-2 applicability, **and** no concerns for COSMIN Box 8
Moderate evidence	Provided by at least two studies with very good quality, defined as ≥ 3 good quality-scores (+) for QUADAS-2 Risk of bias, **and** 3 good-quality scores (+) for QUADAS-2 applicability, **and** no important concerns for COSMIN Box 8
Limited evidence	Provided by one study with very good quality, defined as ≥ 3 good quality-scores (+) for QUADAS-2 Risk of bias, **and** 3 good-quality scores (+) for QUADAS-2 applicability, **and** no important concerns for COSMIN Box 8, **or** at least two studies with good quality, defined as ≥ 2 good quality-scores (+) for QUADAS-2 Risk of bias, **and** ≥ 2 good-quality scores (+) for QUADAS-2 applicability, **and** no important concerns for COSMIN Box 8
Insufficient evidence	In the case that eligible studies do not meet the criteria for one of the above stated levels of evidence
No evidence	In the case of no eligible studies

For all extracted diagnostic accuracy data, the diagnostic evidence was given by the Youden index, which was calculated by deducting 1 from the sum of the sensitivity and specificity [15, 16]. The Youden index was interpreted as weak (< 0.4), moderate (≥ 0.4 and < 0.7), high (≥ 0.7 and < 0.9), and very high (≥ 0.9) as an indicator of the diagnostic value of measurement instruments. Diagnostic evidence was classified as conflicting when the Youden index differed ≥ 0.4 within or between studies.

Diagnostic accuracy data were interpreted as indicators of the index measurement instrument, not the reference measurement instrument. Index instruments could be experimental, while reference measurement instruments were commonly used clinically in the absence of a clear gold standard. Applicability of reference instruments was evaluated during quality assessment.

## Results

### Included studies

**Fig. 1:**
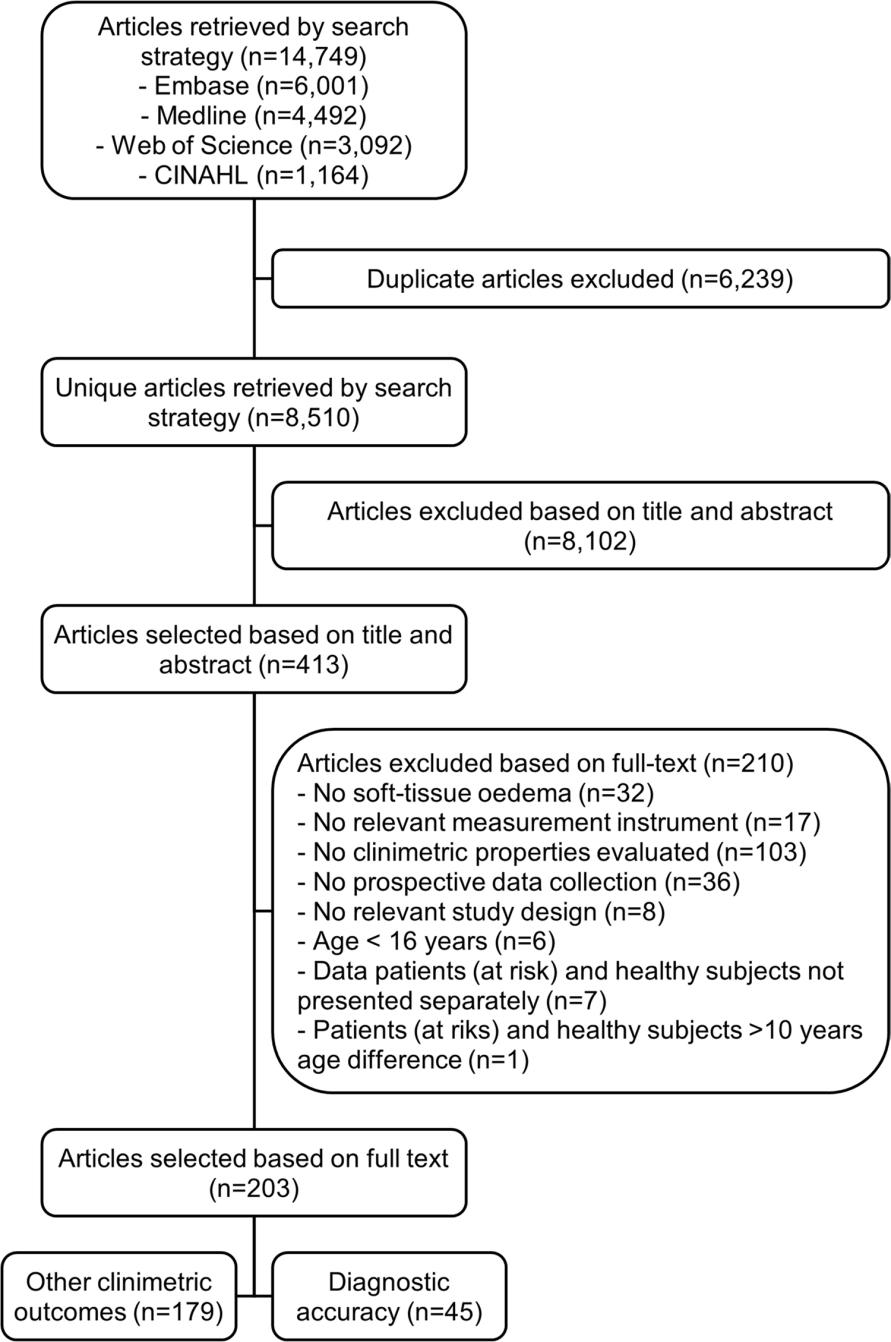
Study selection flowchart

The search identified 8,510 unique articles, with 413 eligible for full-text assessment. Of these, 210 articles were excluded based on the eligibility criteria, and 179 articles assessed clinimetric outcome measures other than diagnostic accuracy, which will be addressed in subsequent reviews. Ultimately, 45 articles on diagnostic accuracy were included in this review, as detailed in the study selection flowchart (Fig. 1).

### Study characteristics

The characteristics of the included studies are presented in Table 2, sorted by index and reference measurement instruments. An overview of the identified measurement instruments is provided in Table 3.

**Table 2:** Study characteristics of included articles

Author, year	Index measurement instrument	Reference measurement instrument	Diagnose	Versus	Diagnostic classification system/threshold	Body part		Population			Measurement instrument specification and threshold		Diagnostic accuracy		
								**Previous diagnosis**	***N* (%F)**	**Age; mean ± SD median (range)**			**Sensitivity**	**Specificity**	**Youden index**
Wiser et al*.* [31], 2020	LSG	Perometry	All stages LE	No LE	Interlimb volume difference > 10%	Upper extremities unilateral		Various	118 (98)	54 ± 11	Presence of dermal backflow or lack of radiotracer uptake after 3 h		0.88	0.41	0.29
Sampathirao et al*.* [58], 2021	LSG	ISL consensus criteria 2020	LE	No LE	ISL consensus criteria 2020	Lower extremities		Various	LE: 86 (43) No LE: 9 (56)	LE: (Range = 18–78) No LE: (Range = 20–45)	Nodal uptake ≤ 22.18%		1.00	0.80	0.80
Keo et al*.* [60], 2013	Fluorescence lymphography	Lymphedema Framework International consensus 2006	LE	No LE	Lymphedema Framework International consensus 2006	Lower extremities		Not specified	LE: 89 (66) No LE: 82 (74)	LE: 47 (IQR = 29–56) No LE: 48 (IQR = 37–58)	Maximum spread of dye	≥ 8 mm	0.94	0.28	0.22
												≥ 10 mm	0.91	0.40	0.31
												≥ 12 mm	0.87	0.64	0.51
												≥ 14 mm	0.79	0.83	0.62
												≥ 15 mm	0.78	0.83	0.61
												≥ 16 mm	0.72	0.84	0.56
Keo et al*.* [59], 2015^a^	Fluorescence lymphography	Lymphedema Framework International consensus 2006	LE	No LE	Lymphedema Framework International consensus 2006	Lower extremities		Not specified	70 (66)	45 (IQR = 27–56)	Maximum spread of dye	≥ 10 mm	0.94	0.51	0.45
												≥ 12 mm	0.94	0.79	0.73
												≥ 14 mm	0.92	0.86	0.78
												≥ 16 mm	0.76	0.89	0.65
Aldrich et al*.* [61], 2022	Fluorescence lymphography	Perometry	Mild to moderate LE	No LE	Relative volume change ≥ 5% to baseline	Upper extremities		BC	42 (100)	54 (28–68)	Dermal backflow present		0.97	0.50	0.47
Berlit et al*.* [35], 2012	SF-BIA	Tape measurements	LE	No LE	NR	Upper extremities		BC unilateral	33 (100)	59.9 ± 9.3	Resistance, threshold NR		0.75	0.86	0.61
											Phase angle, threshold NR		0.75	0.83	0.58
Berlit et al*.* [36], 2013^b^	SF-BIA	Tape measurements	LE	No LE	NR	Upper extremities		BC unilateral	LE: 7 (100) No LE: 35 (100)	LE: 60.3 ± 10.6 No LE: 60.4 ± 11.3	Resistance, threshold NR		0.86	0.97	0.83
Lim et al. [41], 2019	SF-BIA	Tape measurements	LE	No LE	> 2 cm interlimb difference	Upper extremities		BC unilateral	LE: 22 (100) No LE: 206 (100)	LE: 56.3 ± 12.0 No LE: 52.8 ± 9.7	5 kHz, interlimb ratio > 1.047		0.63	0.95	0.58
Dylke et al*.* [17], 2016	MF-BIA	LSG	Dermal backflow score 1 and 2	Dermal backflow score 0	0: no dermal backflow 1: small area or localized backflow 2: circumferential dermal backflow in < 50% of the forearm 3: circumferential dermal backflow in > 50% of the forearm	Upper extremities		BC unilateral	LE: 68 (100) At risk: 6 (100) No LE: 13 (100)	LE: 60.7 ± 11.1 At risk: 50.5 ± 7.5 No LE: 55.9 ± 17.6	Impedance interlimb ratio	3SD	0.96	0.67–0.72	0.63–0.68
												2SD	0.93	0.81–0.87	0.74–0.80
Pichonnaz et al*.* [29], 2015	MF-BIA	MF-BIA (preoperative)	Postoperative (TKA) swelling	No swelling	Percentage difference between the healthy and involved limb (threshold NR)	Lower extremities (unilateral)		Osteoarthritis	24 (50)	69.5 ± 9.7	Postoperative day 2, interlimb difference > 13.4%		0.96	0.96	0.92
											Postoperative day 8, interlimb difference > 13.8%		1.00	0.96	0.96
Bundred et al*.* [37], 2015	MF-BIA	Perometry	LE	No LE	> 10% volume increase from baseline	Upper extremities		BC	612 (100)	55 (24–90)	≥ 10 units difference from baseline		0.73	0.84	0.57
Wiser et al*.* [31], 2020	MF-BIA	Perometry	All stages LE	No LE	Interlimb volume difference > 10%	Upper extremities unilateral		Various	118 (98)	54 ± 11	L-Dex > 10		0.91	0.76	0.67
Cornish et al*.* [38], 2001	MF-BIA	Tape measurements	Early LE	No LE	> 0.139 volume ratio from baseline	Upper extremities		BC unilateral	102 (NR)	51 (25–82)	> 0.102 ratio from baseline		1.00	0.98	0.98
Fu et al*.* [39], 2013	MF-BIA	Tape measurement + clinical assessment + self-report	LE	At risk	Tape measurement > 200 ml interlimb difference	Upper extremities		BC unilateral	LE: 42 (100) At risk: 148 (100)	LE: 58.0 ± 10.7 At risk: 55.8 ± 11.6	L-Dex interlimb ratio	> + 10	0.66	0.95	0.61
												> + 7.1	0.80	0.90	0.70
Barrio et al*.* [34], 2015	MF-BIA	WV	LE	No LE	> 10% interlimb difference	Upper extremities		BC unilateral	186 (100)	60 (27–83)	L-Dex interlimb ratio > 10		0.75	0.93	0.68
Brandini Da Silva Tozzo et al*.* [21], 2023	MF-BIA	WV	LE	No LE	Interlimb volume difference of ≥ 200 mL	Upper extremities		BC	462 (100)	57 ± 9	L-Dex interlimb ratio	≥ 10	0.44	0.95	0.39
												≥ 7.35	0.57	0.91	0.48
												≥ 6.5	0.57	0.89	0.46
												≥ 1.35	0.74	0.67	0.41
Lahtinen et al*.* [40], 2015	MF-BIA	2 of the following 3: WV, clinical assessment (palpation), self-report	LE	No LE	WV ≥ 5% interlimb difference	Upper extremities		BC unilateral	100 (100)	57.4 ± 11.5	Interlimb ratio 1.066 nondominant arm and 1.139 dominant arm		0.42	0.94	0.36
Thomis et al. [33], 2020	TDC measurements	Fluorescence lymphography	Dermal backflow stage I-V	No dermal backflow	Dermal backflow stage: I: splash pattern II: stardust pattern proximally to the olecranon III: stardust pattern exceeds olecranon IV: stardust pattern whole arm V: diffuse pattern	Upper extremities (unilateral)	Hand	BC	45 (NR)	61.3 ± 9.9	Water content interlimb ratio ≥ 1.2		0.65	0.90	0.55
							Ventral forearm						0.81	0.67	0.48
							Dorsal forearm						0.78	0.75	0.53
							Elbow						0.85	0.11	-0.04
							Ventral upper arm						0.67	0.85	0.52
							Dorsal upper arm						0.72	0.67	0.39
							Shoulder						0.33	0.98	0.31
							Overall						0.75	0.75	0.50
Thomis et al*.* [30], 2022^c^	TDC measurements	Fluorescence lymphography	Dermal backflow stage I-IV	No dermal backflow	Dermal backflow stage: 0: No I: splash pattern II: stardust pattern III: diffuse pattern IV: no transport	Upper extremities	All regions	BC unilateral	128 (99)	56.7 ± 12.2	Water content interlimb ratio ≥ 1.2		0.36	0.92	0.28
							Ventral upper arm						0.38	0.91	0.29
Bakar et al*. *[42], 2018	TDC measurements	Tape measurements	Grade 1–3 LE	Grade 0 LE	Circumference interlimb difference Grade 1: 5–10% Grade 2: 10–30% Grade 3: > 30%	Upper extremities		BC unilateral	LE: 31 (100) No LE: 32 (100)	LE: 54.5 ± 13.0 No LE: 53.3 ± 12.9	Local tissue water interlimb ratio ≥ 1.20		0.65	0.94	0.59
Lahtinen et al*. *[40], 2015	TDC measurements	2 of the following 3: WV, clinical assessment (palpation), self-report	LE	No LE	WV ≥ 5% interlimb difference	Upper extremities		BC unilateral	100 (100)	57.4 ± 11.5	Interlimb ratio 1.45 upper arm and 1.30 forearm		0.66	0.84	0.50
Riches et al*. *[43], 2023	TDC measurements	Clinical assessment	LE	No LE	Pitting oedema present ≥ 1 breast quadrant	Breast		BC	89 (NR)	61.1 ± 9.6	Interbreast ratio > 1.34		0.88	0.80	0.68
Liu et al*. *[27], 2022	TDC measurements	ISL consensus criteria 2016	Stage 0 LE	No LE	ISL consensus criteria 2016	Upper extremities		BC unilateral	69 (100)	54.4 ± 9.3	Interlimb ratio ≥ 1.2		0.44	NR	NA
			Stage 1 LE	No LE									0.52	NR	NA
			Stage 2 LE	No LE									0.96	NR	NA
Dylke et al*. *[17], 2016	Perometry	LSG	Dermal backflow score 1 and 2	Dermal backflow score 0	0: no dermal backflow 1: small area or localized backflow 2: circumferential dermal backflow in < 50% of the forearm 3: circumferential dermal backflow in > 50% of the forearm	Upper extremities		BC unilateral	LE: 68 (100) At risk: 6 (100) No LE: 13 (100)	LE: 60.7 ± 11.1 At risk: 50.5 ± 7.5 No LE: 55.9 ± 17.6	Whole arm volume	3SD, truncated cone	1.00	0.57	0.57
												2SD, truncated cone	1.00	0.81	0.81
												3SD, perometry cut-off	1.00	0.62	0.62
												2SD, perometry cut-off	1.00	0.78	0.78
												200 ml difference	0.96	0.78	0.74
												10% increase	0.96	0.78	0.74
											Circumferences	3SD, single elevation	1.00	0.81	0.81
												3SD, 2 + elevation	1.00	0.58	0.58
												2SD, single elevation	0.96	0.94	0.90
												2SD, 2 + elevation	1.00	0.77	0.77
												3SD, SOAC	1.00	0.64	0.64
												2SD, SOAC	1.00	0.77	0.77
												Single 2 cm difference	1.00	0.85	0.85
												2 + 2 cm difference	1.00	0.65	0.65
												SOAC: 5 cm difference	1.00	0.77	0.77
Jeffs and Purushotham [18], 2016	Perometry	Clinical assessment	LE	No LE	Presence of one or more symptoms: decreased visibility of veins, increased thickness of skin and subcutis, fullness of tissues or smoothing of natural limb contours, pitting oedema	Upper extremities		BC	40 (100)	62 (IQR 55–66)	≥ 10% interlimb difference		0.17	1.00	0.17
Hayes et al*. *[24], 2005	Tape measurements	MF-BIA	LE	No LE	Interlimb ratio > 3SD	Upper extremities		BC unilateral	176 (100)	54 ± 10	Sum of arm circumference interlimb difference	> 5 cm	0.35	0.89	0.24
												> 10%	0.05	1.00	0.05
Hayes et al*. *[25], 2008^d^	Tape measurements	MF-BIA	LE	No LE	Interlimb ratio > 3SD	Upper extremities		BC unilateral	211 (100)	54 ± 10	Sum of arm circumference interlimb difference > 5 cm		0.42	0.88	0.30
Wiser et al*. *[31], 2020	Tape measurements	Perometry	All stages LE	No LE	Interlimb volume difference > 10%	Upper extremities unilateral		Various	118 (98)	54 ± 11	Circumference difference > 2 cm		0.83	0.85	0.68
Asim et al*. *[19], 2012	Tape measurements	Tape measurements	Moderate LE	No or mild LE	Lymphedema Framework International consensus 2006; volume ≥ 20% interlimb difference	Upper extremities		BC unilateral	73 (100)	61 ± 11^e^	Circumference interlimb difference	≥ 7.5%	0.83	0.81	0.64
												≥ 10%	0.66	0.89	0.55
												≥ 2 cm	0.66	0.80	0.46
Hidding et al*. *[26], 2018	Tape measurements	Tape measurements	LE	No LE	Volume ≥ 10% interlimb difference	Upper extremities		BC unilateral	51 (98)	51.3 ± 8.5	Circumference > 4% interlimb difference		0.85	0.85	0.70
Bland et al*. *[20], 2003	Tape measurements	Tape measurements with clinical assessment specialist	LE	No LE	> 10% increase in volume from baseline or > 1 cm increase in circumference from baseline, both with confirmation from a lymphoedema specialist (method not specified)	Upper extremities	Elbow	BC	LE: 38 (100) No LE: 52 (100)	LE: 54.8 ± 13.4 No LE: 54.4 ± 10.3	Circumference increase from baseline	> 5%	0.80	0.71	0.51
												> 10%	0.37	0.92	0.29
												> 2 cm	0.59	0.85	0.44
							Any site					> 5%	0.91	0.46	0.37
												> 10%	0.49	0.81	0.30
												> 2 cm	0.70	0.76	0.46
Pichonnaz et al*. *[29], 2015	Tape measurements	Tape measurements (preoperative)	Postoperative (TKA) swelling	No swelling	% difference between the healthy and involved limb (threshold NR)	Lower extremities (unilateral)		Osteoarthritis	24 (50)	69.5 ± 9.7	Postoperative day 2	Volume, interlimb difference > 6.1%	0.83	0.79	0.62
												Circumference, interlimb difference > 5.6%	0.92	0.83	0.75
											Postoperative day 8	Volume, interlimb difference > 7.7%	0.92	0.92	0.84
												Circumference, interlimb difference > 5.8%	0.96	0.88	0.84
Brandini Da Silva Tozzo et al. [21]*,* 2023	Tape measurements	WV	LE	No LE	Interlimb volume difference of ≥ 200 mL	Upper extremities		Breast cancer	462 (100)	57 ± 9	Interlimb difference ≥ 2 cm		0.85	0.83	0.68
Godoy et al*. *[23], 2007	Tape measurements	WV	LE	No LE	≥ 200 mL interlimb difference	Upper extremities		BC unilateral^f^	90 (100)	54.8 ± 11.7	Interlimb difference	≥ 2 cm	0.90	0.72	0.62
					≥ 100 mL interlimb difference							≥ 2 cm	0.87	0.69	0.56
					≥ 10% interlimb difference							≥ 10%	0.73	0.78	0.51
Furlan et al. [22], 2021	Tape measurements	WV	LE	No LE	Interlimb volume difference of ≥ 200 mL	Upper extremities		BC unilateral	85 (NR)	21% < 55y 79% > 55y	Interlimb difference ≥ 2 cm	for two contiguous points	0.53	0.99	0.52
												any point	0.63	0.92	0.55
Lopez Penha et al*. *[28], 2011	Tape measurements	WV	LE	No LE	> 299 mL interlimb difference	Upper extremities		BC	145 (100)	55 (33 -86)^g^	Circumference interlimb difference > 2 cm		0.82	0.73	0.55
											Sum of circumferences interlimb difference > 5 cm		0.82	0.90	0.72
Liu et al*. *[27], 2022	Tape measurements	ISL consensus criteria 2016	Stage 0	No LE	ISL consensus criteria 2016	Upper extremities		BC unilateral	69 (100)	54.4 ± 9.3	Interlimb difference circumference ≥ 2 cm at any point, or volume ≥ 200 mL and/or ≥ 10%		0.00	NR	NA
			Stage 1 LE	No LE									1.00	NR	NA
			Stage 2 LE	No LE									1.00	NR	NA
Thomis et al*. *[30], 2022^c^	Tape measurements (arm) and water volumetry (hand)	Fluorescence lymphography	Dermal backflow stage I-IV	No dermal backflow	Dermal backflow stage: 0: No I: splash pattern II: stardust pattern III: diffuse pattern IV: no transport	Upper extremities	All regions	BC unilateral	128 (99)	56.7 ± 12.2	Interlimb volume difference	≥ 3%	0.60	0.86	0.46
												≥ 5%	0.46	0.92	0.38
							Ventral upper arm					≥ 3%	0.67	0.65	0.32
												≥ 5%	0.51	0.18	-0.31
Thomis et al*. *[33], 2020	WV	Fluorescence lymphography	Dermal backflow stage I-V	No dermal backflow	Dermal backflow stage: I: splash pattern II: stardust pattern proximally to the olecranon III: stardust pattern exceeds olecranon IV: stardust pattern whole arm V: diffuse pattern	Upper extremities (unilateral)	Hand	BC	45 (NR)	61.3 ± 9.9	Interlimb difference ≥ 5%		0.85	0.79	0.64
							Ventral forearm						0.94	0.56	0.50
							Dorsal forearm						0.97	0.75	0.72
							Elbow						0.85	0.16	0.01
							Ventral upper arm						0.94	0.22	0.16
							Dorsal upper arm						1.00	0.26	0.26
							Overall						0.93	0.39	0.32
Brandini Da Silva Tozzo et al*. *[21], 2023	WV (indirect)	WV (direct)	LE	No LE	Interlimb volume difference of ≥ 200 mL	Upper extremities		BC	462 (100)	57 ± 9	Interlimb difference	≥ 200 ml	0.66	0.96	0.62
Lu et al*. *[32], 2014	WV	ISL consensus criteria 2009	Stage 1 LE	Stage 0 LE	ISL consensus criteria 2009	Lower extremities (unilateral)	Calf	GC: cervical or endometrial	LE stage 0: 25 (100) 1: 22 (100) 2: 28 (100) 3: 20 (100)	Median = 57	≥ 10%		0.58	0.97	0.55
											Interlimb volume difference (56 ml)		0.86	0.76	0.62
			Stage 2 LE	Stage 1 LE							Volume (2620 ml)		0.68	0.71	0.39
											Interlimb volume difference (364 ml)		0.84	0.86	0.70
			Stage 3 LE	Stage 2 LE							Volume (3450 ml)		0.95	0.87	0.82
											Interlimb volume difference (1033 ml)		0.90	0.87	0.77
Koo et al*. *[44], 2019	CT	LSG	LSG Stage IV	LSG Stages I, II and III	Stage 1: normal Stage 2: visible, but dysfunctional epifascial lymph nodes Stage 3: dermal backflow and no visible epifascial lymph nodes Stage 4: visible dermal backflow and no epifascial or subfascial lymph nodes	Upper and lower extremities		Various	24 (100)	57.5 ± 13.4	Interlimb ratio number of pixels of tissue layer between skin and muscle 17.57		0.78	0.60	0.38
			LSG Stages III and IV	LSG Stages I and II									0.75	0.56	0.31
Li et al*. *[45], 2015^ h^	MRI	ISL consensus criteria 2009	Stage 1 LE	Stage 0 LE	ISL consensus criteria 2009	Lower extremities (unilateral)	Calf	GC: uterine	LE stage 0: 22 (100) 1: 15 (100) 2: 38 (100) 3: 19 (100)	Median = 57	Total soft tissue thickness (105.4 mm)		0.60	0.82	0.42
											Interlimb difference total soft tissue thickness (5.65 mm)		0.60	0.77	0.37
											Subcutaneous tissue thickness (23.7 mm)		0.67	0.96	0.63
											Interlimb difference subcutaneous tissue thickness (3.45 mm)		0.93	0.82	0.75
											Interlimb difference muscle tissue thickness (0.10 mm)		0.73	0.64	0.37
			Stage 2 LE	Stage 1 LE							Total soft tissue thickness (111.05 mm)		0.71	0.80	0.51
											Interlimb difference total soft tissue thickness (13.85 mm)		0.84	0.80	0.64
											Subcutaneous tissue thickness (30.6 mm)		0.76	0.73	0.49
											Interlimb difference subcutaneous tissue thickness (11.1 mm)		0.84	0.87	0.71
											Interlimb difference muscle tissue thickness (0.90 mm)		0.61	0.60	0.21
			Stage 3 LE	Stage 2 LE							Total soft tissue thickness (124.45 mm)		0.90	0.82	0.72
											Interlimb difference total soft tissue thickness (31.63 mm)		0.84	0.90	0.74
											Subcutaneous tissue thickness (46.95 mm)		0.95	0.90	0.85
											Interlimb difference subcutaneous tissue thickness (29.3 mm)		0.95	0.92	0.87
											Interlimb difference muscle tissue thickness (0.65 mm)		0.47	0.40	-0.13
			Stage 1 LE	Stage 0 LE			Thigh				Total soft tissue thickness (133.25 mm)		0.60	0.59	0.19
											Interlimb difference total soft tissue thickness (7.8 mm)		0.73	0.82	0.55
											Subcutaneous tissue thickness (37.05 mm)		0.73	0.73	0.46
											Interlimb difference subcutaneous tissue thickness (3.65 mm)		0.73	0.73	0.46
											Interlimb difference muscle tissue thickness (0.05 mm)		0.60	0.59	0.19
			Stage 2 LE	Stage 1 LE							Total soft tissue thickness (144.60 mm)		0.73	0.60	0.33
											Interlimb difference total soft tissue thickness (19.85 mm)		0.76	0.72	0.48
											Subcutaneous tissue thickness (46.9 mm)		0.66	0.60	0.26
											Interlimb difference subcutaneous tissue thickness (15.2 mm)		0.76	0.60	0.36
											Interlimb difference muscle tissue thickness (4.55 mm)		0.64	0.60	0.24
			Stage 3 LE	Stage 2 LE							Total soft tissue thickness (158.35 mm)		0.79	0.68	0.47
											Interlimb difference total soft tissue thickness (44.45 mm)		0.74	0.87	0.61
											Subcutaneous tissue thickness (62.3 mm)		0.74	0.68	0.42
											Interlimb difference subcutaneous tissue thickness (38.85 mm)		0.68	0.87	0.55
											Interlimb difference muscle tissue thickness (7.65 mm)		0.63	0.66	0.29
Lu et al*. *[32], 2014	MRI	ISL consensus criteria 2009	Stage 1 LE	Stage 0 LE	ISL consensus criteria 2009	Lower extremities (unilateral)	Calf	GC: cervical or endometrial	LE stage 0: 25 (100) 1: 22 (100) 2: 28 (100) 3: 20 (100)	Median = 57	Total soft tissue thickness (970 mm)		0.74	0.72	0.46
											Interlimb difference total soft tissue thickness (4.6 mm)		0.71	0.92	0.63
											Subcutaneous tissue thickness (18.1 mm)		0.93	0.84	0.77
											Interlimb difference subcutaneous tissue thickness (3.5 mm)		0.96	1.00	0.96
			Stage 2 LE	Stage 1 LE							Total soft tissue thickness (1111 mm)		0.70	0.79	0.49
											Interlimb difference total soft tissue thickness (13.9 mm)		0.84	0.93	0.77
											Subcutaneous tissue thickness (29.2 mm)		0.78	0.71	0.49
											Interlimb difference subcutaneous tissue thickness (11.5 mm)		0.88	0.94	0.82
			Stage 3 LE	Stage 2 LE							Total soft tissue thickness (129.6 mm)		0.84	0.89	0.73
											Interlimb difference total soft tissue thickness (31.7 mm)		0.84	0.89	0.73
											Subcutaneous tissue thickness (48.2 mm)		0.90	0.92	0.82
											Interlimb difference subcutaneous tissue thickness (29.3 mm)		0.90	0.97	0.87
Wang et al*. *[46], 2018	MRI	ISL consensus criteria 2013	Stage 1 LE	Stage 0 LE	ISL consensus criteria 2013	Lower extremities		Various	138 (NR)	NR	Total area soft-tissue (6180 mm^2^)		0.76	0.43	0.19
											Difference total area soft-tissue (306 mm^2^)		0.76	0.64	0.40
											Water area soft-tissue (17 mm^2^)		1.00	1.00	1.00
			Stage 2 LE	Stage 1 LE							Total area soft-tissue (7006.5 mm^2^)		0.75	0.59	0.34
											Difference total area soft-tissue (1281 mm^2^)		0.89	0.91	0.80
											Water area soft-tissue (913 mm^2^)		0.81	0.94	0.75
			Stage 3 LE	Stage 2 LE							Total area soft-tissue (120,200 mm^2^)		0.54	0.85	0.39
											Difference total area soft-tissue (4809 mm^2^)		0.86	0.89	0.75
											Water area soft-tissue (10479.5 mm^2^)		0.72	0.75	0.47
Wiser et al*. *[31], 2020	MRA	Perometry	All stages LE	No LE	Interlimb volume difference > 10%	Upper extremities unilateral		Various	118 (98)	54 ± 11	Fluid accumulation		0.94	0.44	0.38
											Fat hypertrophy		0.96	0.64	0.60
Lee et al*. *[53], 2020	US	LSG	Stage 1 or 2 LE	Stage 0 LE	Stage 0: normal Stage 1: partial lymphatic obstruction Stage 2: total lymphatic obstruction	Lower extremities (unilateral)		Not specified	60 (90)	59.0 ± 11.7	Evenlope amplitude, threshold NR		0.70	0.48	0.18
											Nakagami parameter, threshold NR		0.70	0.75	0.45
											Shannon entropy, threshold NR		0.95	0.88	0.83
Chan et al*. * [47], 2018	US	LSG	Lymphatic obstruction (partial and total)	No lymphatic obstruction	No: lymph nodes and lymphatic collectors are well visualized and without dermal backflow Partial: either decreased visualization of proximal lymph nodes or dermal backflow Total: absence of lymphatic collector and proximal lymph nodes and dermal backflow	Upper (N = 19) and lower extremities (N = 45)		Cancer (N = 56) or none (primary LE, N = 8)	64 (89)	58.4 ± 11.9	Cutaneous shear wave velocity > 2.10 m/s		0.83	0.86	0.69
											Subcutaneous shear wave velocity > 1.43 m/s		0.80	0.70	0.50
Hara and Mihara [52], 2021	US	Fluorescence lymphography	All stages LE	No LE	Presence dermal backflow	Lower extremities		GC	14 (100)	Mean 59.7 (range 48–84)	Presence dilated or sclerotic lymphedema vessels		0.43	0.94	0.37
Devoogdt et al*. *[48], 2014	US	Tape measurements	Mild LE	No LE	> 5% volume interlimb difference	Upper extremities	Wrist	BC unilateral	42 (NR)	54.9 ± 11.4	Thickness subcutis (> 20% interlimb difference)		0.40	0.93	0.33
											Echogenicity cutis (disturbed)		1.00	0.75	0.75
											Echogenicity subcutis (disturbed)		0.33	0.93	0.26
							Ventral				Thickness cutis (> 0.3 mm interlimb difference)		0.33	0.93	0.26
											Thickness subcutis (> 20% interlimb difference)		0.67	0.59	0.26
											Echogenicity cutis (disturbed)		0.20	0.96	0.14
											Echogenicity subcutis (disturbed)		0.13	0.96	0.09
							Dorsal				Thickness cutis (> 0.3 mm interlimb difference)		0.33	0.93	0.23
											Thickness subcutis (> 20% interlimb difference)		0.20	0.63	-0.17
											Echogenicity cutis (disturbed)		0.27	1.00	0.27
											Echogenicity subcutis (disturbed)		0.27	0.93	0.20
							Biceps				Thickness cutis (> 0.3 mm interlimb difference)		0.14	0.86	0.00
											Thickness subcutis (> 20% interlimb difference)		0.24	0.81	0.05
											Echogenicity cutis (disturbed)		0.10	1.00	0.10
											Echogenicity subcutis (disturbed)		0.14	0.90	0.04
							Triceps				Thickness cutis (> 0.3 mm interlimb difference)		0.25	0.65	-0.10
											Thickness subcutis (> 20% interlimb difference)		0.67	0.67	0.34
											Echogenicity cutis (disturbed)		0.24	1.00	0.24
											Echogenicity subcutis (disturbed)		0.43	0.76	0.19
Erdinç Gündüz et al*.* [49], 2021	US	Tape measurements	Grade 3 LE	Grade 2 LE	ISL consensus criteria 2009; > 5 cm interlimb difference	Upper extremities		BC unilateral	34 (100)	59.8 ± 10.7	> 0.22 cm interlimb difference		0.80	0.75	0.55
Giray and Yağcı [51], 2019	US	Tape measurements	All stages LE	No LE	> 200 ml interlimb difference	Upper extremities		BC unilateral	45 (NR)	50.8 ± 8.7	Subcutaneous tissue thickness > 0.17 cm interlimb difference		0.79	0.69	0.48
Riches et al*.* [43], 2023	US	Clinical assessment	LE	No LE	Pitting oedema present ≥ 1 breast quandrant	Breast	Lower outer quadrant	BC	89 (NR)	61.1 ± 9.6	Skin thickness	≥ 2.3 mm	0.84	0.87	0.71
							Lower inner quadrant					≥ 2.6 mm	0.79	0.84	0.63
							Upper outer quadrant					≥ 2.5 mm	0.92	0.92	0.84
							Upper inner quadrant					≥ 3.0 mm	0.91	0.93	0.84
Erdogan Iyigun et al*.* [50], 2019	US	ISL 2013 consensus criteria	Stage 2 LE	Stage 1 LE	ISL consensus criteria 2013	Upper extremities		BC unilateral	36 (100)	50.8 (30–69)	Shear wave velocity > 1.78 m/s		0.63	0.65	0.28
Omura et al*.* [54], 2022	US	ISL consensus criteria 2016	Stage ≥ 1 LE	Stage 0 LE	ISL consensus criteria 2016	Lower extremities		NR	ISL stage 0: 54 (89) 1: 18 (89) IIa: 22 (95) IIb: 26 (89)	ISL stage 0: 60 ± 14 1: 57 ± 19 IIa: 63 ± 12 IIb: 68 ± 15	Thickness (threshold NR)	Dermis	0.73	0.91	0.64
												Dermis SD	0.59	0.64	0.23
											% echogenic region (threshold NR)	Hypodermis	0.69	0.73	0.42
											a part of Gaussian form factor (threshold NR)	Dermis	0.45	0.80	0.25
												Dermis, SD	0.64	0.67	0.31
												Dermis, Combination	0.55	0.73	0.28
												Hypodermis	0.54	0.83	0.37
												Hypodermis, SD	0.71	0.61	0.32
												Hypodermis, Combination	0.84	0.49	0.33
											Effective scatter diameter (threshold NR)	Dermis	0.56	0.87	0.43
												Dermis, SD	0.83	0.57	0.40
												Hypodermis	0.61	0.60	0.21
												Hypodermis, SD	0.46	0.74	0.20
											Effective acoustic concentration (threshold NR)	Dermis	0.54	0.93	0.47
												Dermis, SD	0.78	0.76	0.54
												Hypodermis	0.53	0.76	0.29
												Hypodermis, SD	0.59	0.79	0.38
											Homodyned K distribution scatter clustering (threshold NR)	Dermis	0.61	0.57	0.18
												Dermis, SD	0.68	0.59	0.27
												Hypodermis	0.71	0.81	0.52
												Hypodermis, SD	0.84	0.56	0.40
											Homodyned K distribution ratio of coherent to diffuse signal (threshold NR)	Dermis	0.73	0.73	0.46
												Dermis, SD	0.90	0.36	0.26
												Hypodermis	0.61	0.83	0.44
												Hypodermis, SD	0.74	0.57	0.31
											Combination effective scatter diameter and effective acoustic concentration parameters	Dermis	0.71	0.86	0.57
												Hypodermis	0.44	0.93	0.37
											Combination homodyned K parameters	Dermis	0.65	0.91	0.56
												Hypodermis	0.75	0.81	0.56
											Combination effective scatter diameter, effective acoustic concentration and homodyned K parameters	Dermis	0.73	0.89	0.62
												Hypodermis	0.90	0.66	0.56
											Combination thickness effective scatter diameter and effective acoustic concentration parameters	Dermis	0.71	0.96	0.67
											Combination echogenic region and homodyned K parameters	Hypodermis	0.75	0.81	0.56
											Combination thickness, effective scatter diameter, effective acoustic concentration parameters for the dermis and echogenic region and homodyned K parameters for the hypodermis		0.75	0.91	0.66
			Stage ≥ IIa LE	Stage ≤ 1 LE							Thickness (threshold NR)	Dermis	0.88	0.84	0.72
												Dermis SD	0.59	0.59	0.18
											% echogenic region (threshold NR)	Hypodermis	0.80	0.69	0.49
											a part of Gaussian form factor (threshold NR)	Dermis	0.46	0.75	0.21
												Dermis, SD	0.71	0.64	0.35
												Dermis, Combination	0.57	0.67	0.24
												Hypodermis	0.63	0.79	0.42
												Hypodermis, SD	0.79	0.57	0.36
												Hypodermis, Combination	0.91	0.45	0.36
											Effective scatter diameter (threshold NR)	Dermis	0.61	0.79	0.40
												Dermis, SD	0.86	0.49	0.35
												Hypodermis	0.61	0.54	0.15
												Hypodermis, SD	0.54	0.73	0.27
											Effective acoustic concentration (threshold NR)	Dermis	0.70	0.90	0.60
												Dermis, SD	0.86	0.67	0.53
												Hypodermis	0.54	0.69	0.23
												Hypodermis, SD	0.71	0.77	0.48
											Homodyned K distribution scatter clustering (threshold NR)	Dermis	0.50	0.46	-0.04
												Dermis, SD	0.70	0.53	0.23
												Hypodermis	0.86	0.77	0.63
												Hypodermis, SD	0.91	0.50	0.41
											Homodyned K distribution ratio of coherent to diffuse signal (threshold NR)	Dermis	0.79	0.65	0.44
												Dermis, SD	0.91	0.30	0.21
												Hypodermis	0.71	0.78	0.49
												Hypodermis, SD	0.79	0.52	0.31
											Combination effective scatter diameter and effective acoustic concentration parameters	Dermis	0.82	0.78	0.60
												Hypodermis	0.50	0.87	0.37
											Combination homodyned K parameters	Dermis	0.77	0.84	0.61
												Hypodermis	0.89	0.76	0.65
											Combination effective scatter diameter, effective acoustic concentration and homodyned K parameters	Dermis	0.86	0.81	0.67
												Hypodermis	0.98	0.56	0.54
											Combination thickness effective scatter diameter and effective acoustic concentration parameters	Dermis	0.86	0.87	0.73
											Combination echogenic region and homodyned K parameters	Hypodermis	0.86	0.73	0.59
											Combination thickness, effective scatter diameter, effective acoustic concentration parameters for the dermis and echogenic region and homodyned K parameters for the hypodermis		0.89	0.83	0.72
Dylke et al*.* [55], 2018	US	NR	LE	No LE	NR	Breast	Superior	BC	38 (100)	Range = 36–70	Dermal thickness	1.6 mm	0.84	0.91	0.75
												1.9 mm	0.68	0.74	0.42
												2.1 mm	0.60	0.87	0.47
							Medial					2.0 mm	0.90	0.92	0.82
												2.2 mm	0.83	0.97	0.80
												2.5 mm	0.80	0.87	0.67
							Inferior					2.0 mm	0.90	0.92	0.82
												2.1 mm	0.83	0.95	0.78
												2.4 mm	0.80	0.87	0.67
							Lateral					1.6 mm	0.84	0.91	0.75
												1.5 mm	0.88	0.83	0.71
												1.8 mm	0.72	0.74	0.46
Thomis et al*.* [33], 2020	Clinical assessment	Fluorescence lymphography	Dermal backflow stage I-V	No dermal backflow	Dermal backflow stage: I: splash pattern II: stardust pattern proximally to the olecranon III: stardust pattern exceeds olecranon IV: stardust pattern whole arm V: diffuse pattern	Upper extremities (unilateral)	Hand	BC	45 (NR)	61.3 ± 9.9	Pitting doubtful or clearly present		0.54	0.95	0.49
							Ventral forearm						0.83	0.67	0.50
							Dorsal forearm						0.89	0.75	0.64
							Elbow						0.58	0.58	0.16
							Ventral upper arm						0.33	0.85	0.18
							Dorsal upper arm						0.67	0.89	0.56
							Shoulder						0.33	0.91	0.24
							Overall						0.68	0.83	0.51
							Hand				Interlimb difference (increase) in skinfold thickness		0.92	0.58	0.50
							Ventral forearm						0.92	0.33	0.25
							Dorsal forearm						0.92	0.75	0.67
							Elbow						0.96	0.32	0.28
							Ventral upper arm						0.56	0.82	0.38
							Dorsal upper arm						0.89	0.44	0.33
							Shoulder						0.33	0.81	0.14
							Overall						0.87	0.62	0.49
							Hand				Interlimb difference (hard oedema) in elasticity		0.23	1.00	0.23
							Ventral forearm						0.33	0.89	0.22
							Dorsal forearm						0.32	0.88	0.20
							Elbow						0.07	0.95	0.02
							Ventral upper arm						0.06	0.96	0.02
							Dorsal upper arm						0.11	0.85	-0.04
							Shoulder						0.33	1.00	0.33
							Overall						0.22	0.95	0.17
Thomis et al*.* [30], 2022^c^	Clinical assessment	Fluorescence lymphography	Dermal backflow stage I-IV	No dermal backflow	Dermal backflow stage: 0: No I: splash pattern II: stardust pattern III: diffuse pattern IV: no transport	Upper extremities	All regions	BC unilateral	128 (99)	56.7 ± 12.2	Pitting present		0.19	0.98	0.17
											Increased skinfold thickness		0.29	0.97	0.26
							Ventral upper arm				Pitting present		0.14	0.99	0.13
											Increased skinfold thickness		0.29	0.97	0.26
Svensson et al*.* [56], 2020	Clinical assessment	MF-BIA	LE	No LE	Interlimb ratio > 2SD or 3SD	Upper extremities (unilateral)		BC	100 (100)	61.8 ± 10.6	Pitting test, interlimb difference in depth and resolution time		0.92	0.77	0.69
											Forearm pinch test, interlimb difference in tissue texture		0.94	0.75	0.69
											Upper arm pinch test, interlimb difference in tissue texture		0.73	0.77	0.50
Chung et al*.* [57], 2006	Cuff-leak test	Endoscopy	Severe oedema	No severe oedema	Swollen bilateral vocal cords and attachment to opposite side of the larynx	Laryngeal		Not specified	32 (34)	71.3 ± 13.6	Volume < 140 mL		0.89	0.90	0.79

Abbreviations: *BC* breast cancer, *CT* computed tomography, *GC* gynecological cancer, *LE* lymphedema, *LSG* lymphoscintigraphy, *MF-BIA* multi-frequency bioimpedance analysis, *MRA* magnetic resonance angiography, *MRI* magnetic resonance imaging, *NR* not reported, *SF-BIA* single-frequency bioimpedance analysis, *TDC* tissue dielectric constant, *TKA* total knee arthroplasty, *US* ultrasound, *WV* water volumetry

^a^Partly same study population as Keo et al*.* [60]

^b^Partly same study population as Berlit et al*. *[35]

^c^Partly same study population as Thomis et al*. *[33]

^d^Partly same study population as Hayes et al*. *[24]

^e^Data for full group (*N* = 193)

^f^Unilaterality assumed since measurement instrument compares affected to unaffected side

^g^Age at surgery

^h^Partly same study population as Lu et al*. *[32]

**Table 3:**
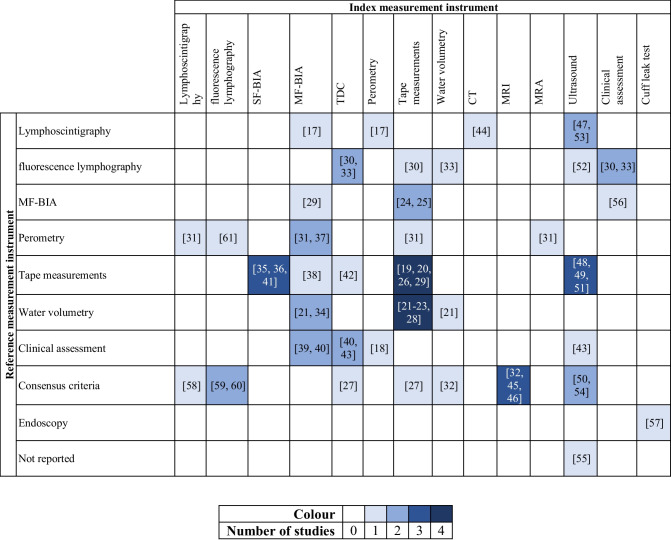
Summary of the index and reference measurement instruments identified for measuring oedema in the included studies

*CT*  computed tomography, *MF-BIA*  multi-frequency bioimpedance analysis, *MRA*  magnetic resonance angiography, *MRI*  magnetic resonance imaging, *SF-BIA*  single-frequency bioimpedance analysis, *TDC*  tissue dielectric constant

The studies evaluated various instruments for diagnosing soft-tissue oedema. Seventeen studies assessed volume measurement instruments, including perometry [17, 18], tape measurements [19–31], and water volumetry [21, 32, 33]. Sixteen studies focused on measurement instruments for tissue characteristics, involving bio-electrical impedance analysis (BIA) [17, 21, 29, 31, 34–41] and tissue-dielectric constant (TDC) [27, 30, 40, 42, 43]. Nineteen studies examined instruments measuring both volume and tissue characteristics, including CT [44], MRI [32, 45, 46], magnetic resonance angiography (MRA) [31], US [43, 47–55], clinical assessment [30, 33, 56], and cuff leak test [57]. Additionally, five studies evaluated the function of the lymphatic system using lymphoscintigraphy [31, 58] and fluorescence microlymphography [59–61].

Reference measurement instruments included volume measurements in 20 studies using perometry [31, 37, 61], tape measurements [19, 20, 26, 29, 35, 36, 38, 41, 42, 48, 49, 51] or water volumetry [21–23, 28, 34]. Tissue characteristics measurements served as reference in three studies using multi-frequency (MF)-BIA [24, 25, 29]. A combination of volume and tissue characteristics measurements was used as reference in 14 studies, involving clinical assessment [18, 39, 40, 43], consensus criteria [27, 32, 45, 46, 50, 54, 58–60] and endoscopy [57]. The lymphatic system function was used as reference in seven studies, using lymphoscintigraphy [17, 44, 47, 53], or fluorescence lymphography [30, 33, 52]. One study did not report the reference instrument [55].

The types of swelling considered were lymphoedema in 44 studies [17–28, 30–61] and postoperative swelling in one study [29]. Secondary lymphoedema arose from (treatment for) various conditions, including breast cancer [17–28, 30, 33–43, 48–51, 55, 56, 61], gynaecological cancer [32, 45, 52], or various causes [31, 44, 46, 47, 58]. In some cases, the cause of lymphoedema was unspecified [53, 54, 57, 59, 60]. The postoperative swelling study focused on total knee arthroplasty for osteoarthritis [29]. Of the 45 included studies, 30 focused on oedema in the upper extremities [17–28, 30, 31, 33–36, 38–42, 48–51, 56, 61], 10 in the lower extremities [29, 32, 45, 46, 52–54, 58–60], 2 in both upper and lower extremities [44, 47], 2 in the breast [43, 55] and 1 in the larynx [57], as illustrated in Table 4. The total number of participants across the studies was 4644, with 94% of the patients being female when gender was reported. Seven studies did not report gender distribution [22, 33, 38, 43, 46, 48, 51]. The mean or median age of participants ranged from 45.0 to 71.3 years.

**Table 4:**
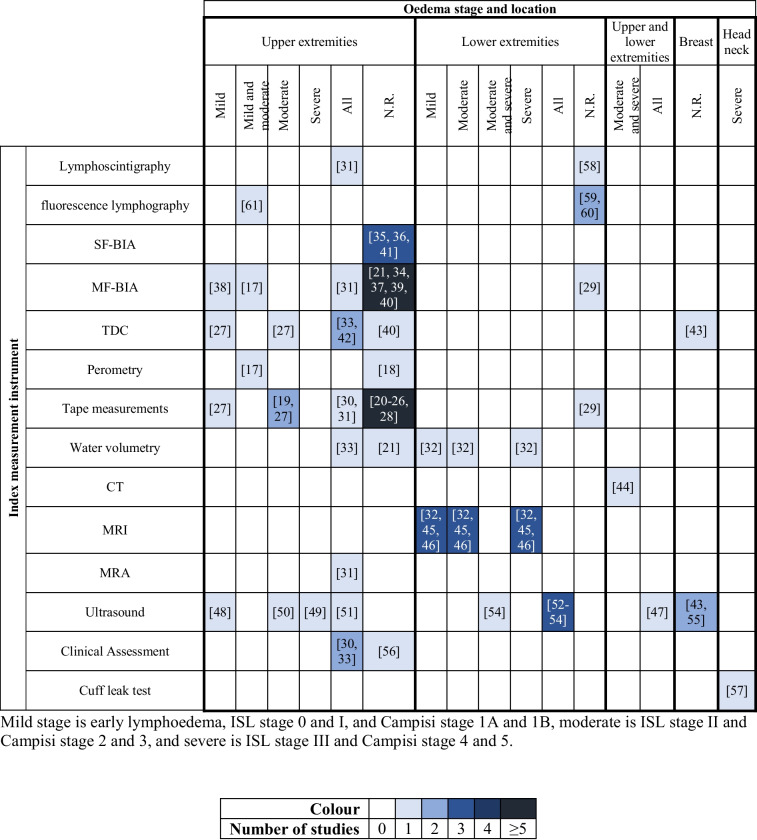
Summary of the oedema location and stage per identified index measurement instruments in the included studies

*CT*  computed tomography, *MF-BIA*  multi-frequency bioimpedance analysis, *MRA*  magnetic resonance angiography, *MRI*  magnetic resonance imaging, *N.R.*  not reported, *SF-BIA*  single-frequency bioimpedance analysis

### Risk of bias within studies

The quality assessment of the included studies is summarized in Table 5, with detailed assessment available in Supplementary information 3. According to QUADAS-2 assessment, no study was free from bias in all domains. Thirty-seven studies (82%) exhibited a high risk of bias in at least one domain [17–22, 24–26, 29–33, 35–39, 41, 43–51, 53–55, 57–61], while eight studies (18%) showed an unclear risk of bias in at least one domain [23, 27, 28, 34, 40, 42, 52, 56]. Common bias sources included unspecified patient enrolment, unspecified blinding, and non-prespecified thresholds. Nevertheless, all studies, except one [55], were considered applicable to the research question. All studies, except one [27], employed very good statistical methods as per COSMIN Box 8.

**Table 5:**
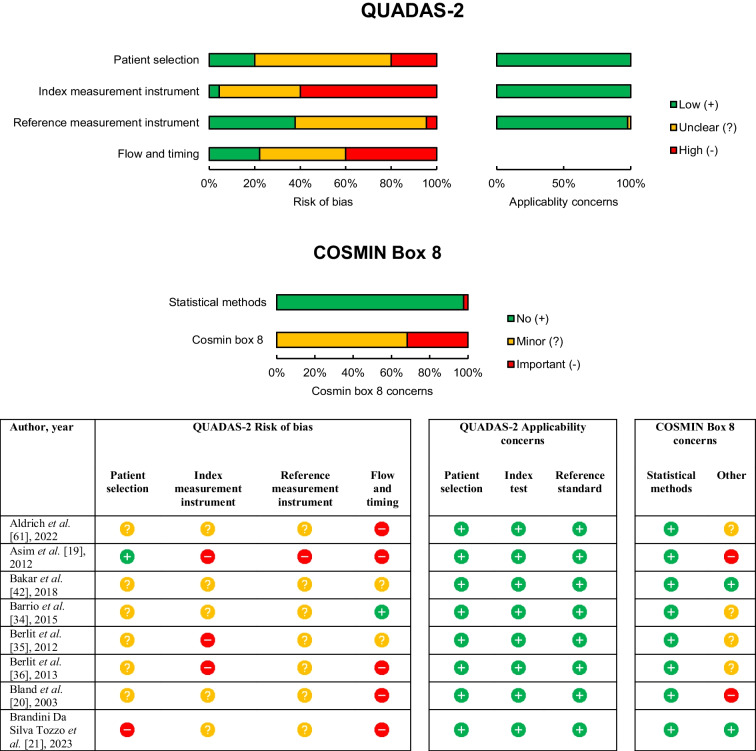

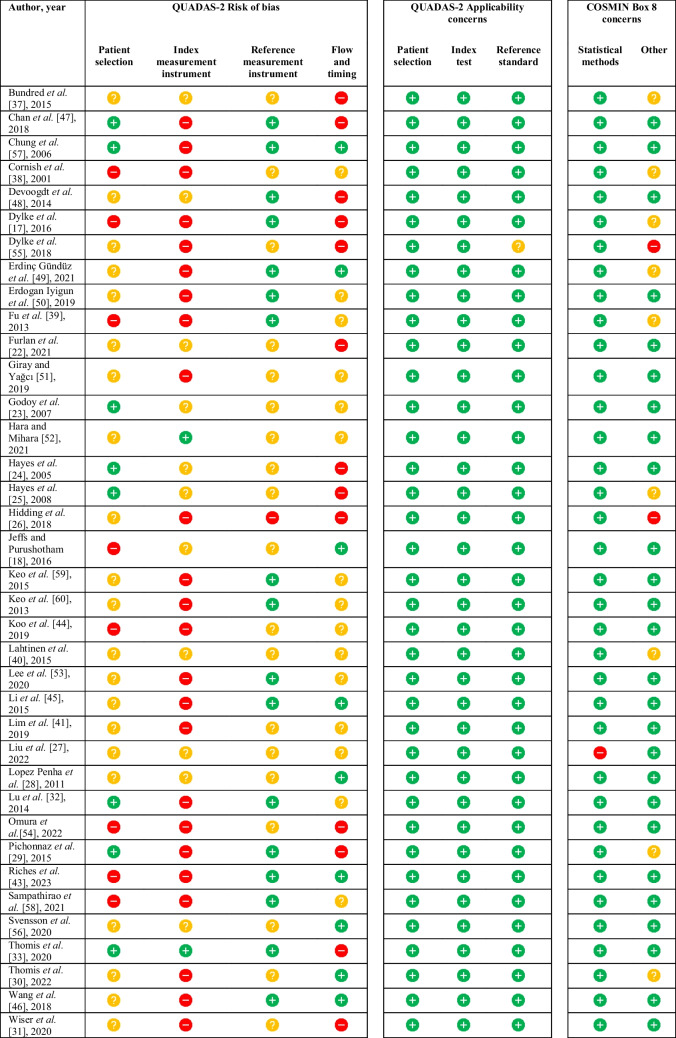
Methodological quality assessment with QUADAS-2 and COSMIN Box 8

### Best-evidence synthesis

Only two studies received three or more good quality scores on the QUADAS-2 risk of bias assessment, limiting the overall level of evidence for index measurements [33, 57]. Limited evidence was found for TDC, water volumetry, MRI, ultrasound, clinical assessment, and cuff leak test. Insufficient evidence was found for lymphoscintigraphy, fluorescence lymphography, SF-BIA, MF-BIA, perometry, tape measurements, CT, and MRA.

### Oedema measurement instruments

#### Instruments assessing lymphatic system function

Lymphoscintigraphy (LSG), using a radioactive tracer, was reported in two studies as an index measurement instrument, demonstrating insufficient evidence with a conflicting diagnostic value (Youden index = 0.29–0.80) [31, 58]. LSG was applied to both upper and lower extremities to quantify lymphoedema by assessing the presence of dermal backflow and the uptake of the radiotracer.

Fluorescence lymphography, e.g. indocyanine green (ICG) lymphography or near-infrared fluorescence imaging (NIRF), was assessed in three studies, although two of these included overlapping populations. Lymphoscintigraphy displayed insufficient evidence with a conflicting diagnostic value (Youden index = 0.22–0.78) for lower extremities, where the diagnosis of lymphoedema was based on the maximum spread of the fluorescent dye [59, 60]. A moderate diagnostic value (Youden index = 0.47) was reported for mild and moderate lymphoedema in upper extremities by measuring dermal backflow [61].

#### Instruments assessing tissue characteristics

Single-frequency (SF) bio-impedance analysis (BIA) was reported in three studies as an index measurement instrument for upper extremities, showing insufficient evidence with a moderate to high diagnostic value (Youden index = 0.58–0.83) [35, 36, 41]. Multi-frequency (MF) BIA, also known as bio-impedance spectroscopy, was evaluated in nine studies. MF-BIA showed insufficient evidence with a conflicting diagnostic value (Youden index = 0.36–0.98) across both upper and lower extremities [17, 21, 29, 31, 34, 37–40]. Notably, for mild lymphoedema in upper extremities, MF-BIA displayed a very high diagnostic value with a Youden index of 0.98, although the risk of bias was unclear to high [38]. Mild and moderate lymphoedema in upper extremities had moderate to high Youden indexes (0.63–0.80) with also an unclear to high risk of bias [17]. In the context of post-operative swelling, MF-BIA exhibited very high diagnostic accuracy (Youden index = 0.92–0.96) by measuring the interlimb difference; however, there was a high risk of bias on two domains of QUADAS-2 [29]. MF-BIA often assessed lymphoedema by evaluating the L-DEX ratio, which compares the impedance of extracellular fluid between the unaffected and affected body parts, with MF-BIA’s ability to distinguish between intracellular and extracellular fluids, in contrast to SF-BIA.

Tissue dielectric constant (TDC) was reported in six studies, presenting limited evidence with a conflicting diagnostic value (Youden index = −0.04 to 0.68) [27, 30, 33, 40, 42, 43]. The Youden index could not be calculated in one study that assessed mild and moderate lymphoedema in the upper extremities [27]. A high-quality study comparing TDC with fluorescence lymphography for all stages of lymphoedema in the upper extremities demonstrated conflicting Youden indexes (−0.04 to 0.52) [33]. Another high-quality study compared TDC with clinical assessment in the breast, showing a moderate Youden index of 0.68 [43]. In all studies, lymphoedema was diagnosed by measuring the water content ratio between affected and unaffected body parts using TDC.

#### Instruments assessing volume

Perometry was evaluated in two studies as an index measurement instrument, demonstrating insufficient evidence with a conflicting diagnostic value (Youden index = 0.17–0.90) in the upper extremities [17, 18]. For the diagnosis of mild and moderate lymphoedema, perometry yielded moderate to very high Youden indexes (0.57–0.90) with an unclear to high risk of bias [17]. The highest Youden index (0.90) was observed when diagnosing oedema using a single elevated circumference, with LSG serving as the reference instrument [17]. In addition to obtaining circumference measurements, volume was also calculated based on the circumference or perometry directly measured volume.

Tape measurements, reported in 13 studies, showed insufficient evidence with a conflicting diagnostic value (Youden index = −0.31 to 0.84) [19–31]. The Youden index could not be calculated in one study that assessed mild and moderate lymphoedema in the upper extremities [27]. For the diagnosis of moderate lymphoedema in the upper extremities, tape measurements yielded moderate Youden indexes (0.46–0.64) when comparing circumferences to calculated volumes, though the studies exhibited a high risk of bias in three domains of the QUADAS-2 [19]. Tape measurements were also applied in the lower extremities. Across the studies, tape measurements were used to evaluate differences between affected and unaffected limbs in 12 studies [19, 21–29, 31], while one study measured differences from the baseline [20]. The method involved measuring various circumferences, and sometimes volume was calculated based on these circumferences.

Water volumetry, evaluated in three studies, displayed limited evidence with a conflicting diagnostic value (Youden index = 0.01–0.82) [21, 32, 33]. In one high-quality study comparing water volumetry with fluorescence lymphography, conflicting Youden indexes (0.01 to 0.72) were reported for the upper extremities [33]. In the lower extremities, water volumetry was used to assess mild, moderate, and severe lymphoedema, yielding weak to high Youden indexes (mild, 0.55–0.62; moderate, 0.39–0.70; severe, 0.77–0.82), with an unclear or high risk of bias in two domains of the QUADAS-2 [32]. All three studies focused on evaluating the volume difference between the affected and unaffected sides. Additionally, one of these studies assessed the diagnostic accuracy of water volumetry using the absolute limb volume, resulting in Youden indexes ranging from 0.39 to 0.82 [32].

#### Imaging techniques assessing both tissue characteristics and volume

Computed tomography (CT) was reported in one study as an index measurement instrument, showing insufficient evidence with a weak diagnostic value (Youden index = 0.31–0.38) for diagnosing moderate and severe lymphoedema in upper and lower extremities [44]. The diagnosis of oedema using CT was based on the ratio of the number of pixels of the tissue layer between skin and muscle between the affected and unaffected sides.

Magnetic resonance imaging (MRI), assessed in three studies, displayed limited evidence with a conflicting diagnostic value (Youden index = −0.13 to 1.00). MRI was used to evaluate mild, moderate, and severe lymphoedema, with each stage showing conflicting Youden indexes (mild, 0.19–1.00; moderate, 0.21–0.82; severe, −0.13 to 0.87) [32, 45, 46]. Notably, very high Youden indexes were observed for diagnosing mild lymphoedema by measuring the difference in subcutaneous tissue thickness between affected and unaffected limbs (Youden index = 0.96) [32] and assessing the absolute water area (Youden index = 1.00) [46]. MRI measurements involved the thickness or area of specific tissue, allowing for the differentiation between water, muscle, subcutaneous tissue, and total soft tissue.

Magnetic resonance angiography (MRA) was evaluated in a single study, providing insufficient evidence with weak to moderate diagnostic value (Youden index = 0.38–0.60) for all stages of lymphoedema in the upper extremities [31]. The diagnosis of lymphoedema using MRI was based on measurements of fluid accumulation and fat hypertrophy.

Ultrasound (US) was evaluated in ten studies, showing limited evidence with a conflicting diagnostic value (Youden index = −0.17 to 0.84) [43, 47–55]. Among these studies, three were of high quality. One high-quality study compared US to clinical assessment and found moderate to high diagnostic value (Youden index = 0.63–0.84) in the breast [43]. Another high-quality study compared US to LSG across all stages of lymphoedema, showing moderate diagnostic value (Youden index = 0.50–0.69) for US in both upper and lower extremities [47]. A third high-quality study compared US to tape measurements for severe lymphoedema in the upper extremities, also demonstrating moderate diagnostic value (Youden index = 0.55) for US [49]. For other stages and locations, the Youden indexes were weak for moderate and severe lymphoedema in the lower extremities (0.18–0.64) [54], conflicting for lymphoedema in the breast (0.42–0.82) [55] and conflicting for mild (−0.17 to 0.75) [48] or moderate lymphoedema (0.28) [50] in the upper extremities. Besides, each of these studies had a risk of bias.

Various outcome parameters were measured using US, with some showing high diagnostic value. For instance, the thickness of the skin yielded Youden indexes as high as 0.84 [43] and the thickness of the dermis reached up to 0.82 [54, 55]. Disturbed echogenicity of the cutis was another parameter with high diagnostic value, achieving a Youden index up to 0.75 [48]. Additionally, some calculated parameters based on algorithmic schemes and mathematical formulas, such as Shannon entropy (Youden index = 0.83) [53] or a combination of various calculated parameters (Youden index up to 0.73) [54], showed strong diagnostic performance. Other outcomes, such as share wave velocity [47, 50], which depends on the tissue density, and the presence of dilated or sclerotic lymphedema vessels [52], demonstrated lower diagnostic value.

#### Other methods assessing both tissue characteristics and volume

Clinical assessment was evaluated in three studies as an index measurement instrument, showing limited evidence with a conflicting diagnostic value (Youden index = −0.04 to 0.69) in the upper extremities [30, 33, 56]. Clinical assessment involved evaluating the presence of pitting, increased skinfold thickness, and deviations in tissue texture. In one high-quality study that compared clinical assessment with fluorescence lymphography, the Youden indexes for the presence of pitting and increased skinfold thickness were weak, ranging from 0.13 to 0.26 [33].

Cuff-leak test was reported once, showing limited evidence with a high diagnostic value (Youden index = 0.79) for laryngeal oedema. Endoscopy was used as the reference instrument [57].

## Discussion

This systematic review aimed to identify measurement instruments for quantitatively diagnosing soft-tissue oedema, including lymphoedema, across any body part and their diagnostic accuracy. The findings highlight both advancements and ongoing challenges in accurately diagnosing lymphoedema.

Our review identified a variety of instruments focused on measuring volume, tissue characteristics, and lymphatic system function. Tape measurements, US, and MF-BIA were the most frequently studied. Index instruments with very high diagnostic value (Youden index ≥ 0.90) included MF-BIA, perometry, and MRI. However, when considering the quality of these studies, evidence is lacking. Limited evidence was found for TDC, water volumetry, MRI, US, clinical assessment, and cuff leak test, while insufficient evidence was found for lymphoscintigraphy, fluorescence lymphography, SF-BIA, MF-BIA, perometry, tape measurements, CT, and MRA. These methodological limitations require cautious interpretation of the current evidence.

Interpretation of evidence is also hampered by the absence of a universal gold standard, resulting in the use of various measurement instruments as reference, making comparisons between studies challenging. The most used reference instruments were tape measurements, consensus criteria, and water volumetry, applied in 12, nine, and five studies, respectively. The frequent use of water volumetry aligns with recommendations from an earlier review suggesting it as the preferred reference standard [23]. The suitability of instruments as a reference depends on the body part being assessed and the stage of lymphoedema. In ISL stage 0, only impaired lymph transport and subtle changes in tissue fluid or composition can be measured, while volumetric measurement becomes more relevant in later stages [2, 9]. Furthermore, a single body part may present with multiple stages simultaneously [2].

Effective lymphoedema management relies on early detection, ideally before irreversible changes such as fibrotic tissue formation occur. The diagnostic accuracy of index measurement instruments for early lymphedema was evaluated for MF-BIA, TDC, tape measurements, water volumetry, MRI, and US in extremities. Diagnostic value was very high for MF-BIA, weak to high for water volumetry, conflicting for MRI and ultrasound, and could not be determined for TDC and tape measurements. However, all evidence was restricted by the limited number of studies.

### Strength and limitations

A key strength of this review is the inclusion of a wide variety of instruments and parameters for diagnosing lymphoedema. However, the inclusion of various instruments and parameters and lack of a uniform and reliable gold (reference) standard precluded meta-analysis and poses a challenge in assessing potential publication bias. This precluded standard methods like funnel plots or Egger’s test for a comprehensive evaluation. Although a best-evidence synthesis was conducted to summarize the overall level of evidence per instrument, it did not account for potential bias in subgroup outcomes for oedema stages or locations.

### Implications for clinical practice

Given the chronic and progressive nature of lymphoedema, early detection is critical for effective management. Despite the extensive range of instruments studied, no single instrument emerged as a definitive gold standard for diagnosing lymphoedema. This finding reflects the multifaceted nature of lymphoedema, which manifests through various physical changes including fluid accumulation and tissue fibrosis. Consequently, the choice of measurement instrument should depend on the specific clinical context and the stage of lymphoedema. However, this review found insufficient evidence on the diagnostic accuracy of specific instruments to recommend their use in particular stages, especially the early stage. Practical tools like tape measurements, consensus criteria, and water volumetry are commonly used because they are inexpensive and easy to use, while advanced imaging techniques may be more appropriate for complex cases (e.g. presence of significant comorbidity) [2] or research settings.

### Recommendations

To enhance the early detection and treatment of lymphoedema, it is essential to focus on several key areas. First, developing standardized protocols for lymphoedema measurement is crucial to reduce variability and improve comparability across studies. This standardization would help in establishing more consistent and reliable diagnostic criteria. In addition, there is a need for comprehensive evaluations through high-quality studies that assess the diagnostic accuracy of measurement instruments. Such studies should aim to fill the gaps in current knowledge and provide robust evidence to support clinical decision-making. Exploring the integration of different measurement techniques could also be beneficial. By combining various tools, healthcare providers may achieve a more accurate and comprehensive assessment of lymphoedema, which could improve diagnosis and management strategies.

In conclusion, this systematic review underscores the complexity of accurately diagnosing lymphoedema. While several measurement instruments are available, there remains a need for more high-quality research to improve standardization. Clinicians must carefully consider the available evidence and the specific clinical context, for example, early detection, when selecting measurement instruments for diagnosing lymphoedema.

## Supplementary Information

Below is the link to the electronic supplementary material.

Supplementary Material 1 (PDF 316 KB)

Supplementary Material 2 (PDF 113 KB)

Supplementary Material 3 (PDF 30.2 KB) 

## Data Availability

No datasets were generated or analysed during the current study.
