# Supplementary material for: The diagnostic accuracy of soft tissue oedema measurements: a systematic review and best-evidence synthesis
Source: Support Care Cancer. 2026 Mar 5;34(3):281. doi: 10.1007/s00520-026-10373-y (PMC12960331; doi:10.1007/s00520-026-10373-y)
Supplement: Supplementary file 1 — Supplementary Material 1 (PDF 316 KB) [file 520_2026_10373_MOESM1_ESM.pdf]

Margje B. Buitenhuis, Elise M. Gane, Janine T. Hidding, Judith D. de Rooij, Wichor M. Bramer, Remco de Bree, Caroline M. Speksnijder, **The diagnostic accuracy of soft tissue oedema measurements: a systematic review and best-evidence synthesis**, *Support Care Cancer.*
Corresponding author: Caroline M. Speksnijder, Department of Oral and Maxillofacial Surgery and Department of Head and Neck Surgical Oncology, University Medical Center Utrecht, Utrecht University, Utrecht, The Netherlands, e-mail: C.M.Speksnijder@umcutrecht.nl

Supplementary information 1. Search string

Embase.com

((('edema'/exp OR lymphedema/exp OR swelling/de) AND (volume/de OR 'quantitative analysis'/de OR 'disease severity assessment'/de OR 'measurement precision'/de)) OR (((lymphedem* OR lymphoedem* OR oedema OR edema OR swelling*) NEAR/6 (size OR measurement* OR volume* OR quanti* OR assess* OR staging OR circumferen*))):ab,ti) AND ('validation study'/de OR 'comparative study'/de OR 'psychometry'/exp OR 'outcome assessment'/exp OR 'observer variation'/exp OR 'interrater reliability'/de OR 'intrarater reliability'/de OR 'health status indicator'/de OR 'reproducibility'/exp OR 'reliability'/de OR 'discriminant analysis'/exp OR 'coefficient of variation'/de OR 'internal consistency'/de OR 'instrument validation'/de OR 'calibration'/de OR 'measurement error'/de OR 'sensitivity and specificity'/de OR (psychometr* OR clinimetr* OR clinometr* OR (outcome NEAR/3 (assessment* OR measure*)) OR (observer* NEAR/3 variation*) OR reproducib* OR reliab* OR unreliab* OR valid* OR coefficient OR homogeneity OR homogeneous OR (internal* NEAR/3 consisten*) OR (cronbach* NEAR/3 (alpha OR alphas)) OR (item NEAR/3 (correlation* OR selection* OR reduction*)) OR agreement OR precision OR imprecision OR (precise* NEAR/3 value*) OR (test NEAR/3 retest) OR (reliab* NEAR/3 (test OR retest)) OR stabilit* OR interrater* OR inter-rater* OR intrarater* OR intra-rater* OR intertester* OR inter-tester* OR intratester* OR intra-tester* OR interobserver* OR inter-observer* OR intraobserver* OR intra-observer* OR intertechnician* OR inter-technician* OR intratechnician* OR intra-technician* OR interexaminer* OR inter-examiner* OR intraexaminer* OR intra-examiner* OR interassay* OR inter-assay* OR intraassay* OR intra-assay* OR interindividual* OR inter-individual* OR intraindividual* OR intra-individual* OR interparticipant* OR inter-participant* OR intraparticipant* OR intra-participant* OR kappa OR kappa-s OR kappas OR repeatab* OR ((replicab* OR repeated) NEAR/3 (measure OR measures OR findings OR result OR results OR test OR tests)) OR generaliza* OR generalisa* OR concordance OR (intraclass NEAR/3 correlation*) OR discriminative OR 'known group' OR (factor NEXT/1 (analys* OR structure*)) OR dimension* OR subscale* OR (multitrait NEAR/3 scaling NEAR/3 (analysis OR analyses))

OR 'item discriminant' OR (interscale NEAR/3 correlation*) OR error OR errors OR ((individual OR interval OR rate OR analysis OR values) NEAR/3 variabil*) OR (uncertainty NEAR/3 (measurement OR measuring)) OR 'standard error of measurement' OR sensitiv* OR responsive* OR (limit NEAR/3 detection) OR 'minimal detectable concentration' OR interpretab* OR ((minimal OR minimally OR clinical OR clinically) NEAR/3 (important OR significant OR detectable) NEAR/3 (change OR difference)) OR (small* NEAR/3 (real OR detectable) NEAR/3 (change OR difference)) OR 'meaningful change' OR 'ceiling effect' OR 'floor effect' OR 'Item response model' OR IRT OR Rasch OR 'Differential item functioning' OR DIF OR 'computer adaptive testing' OR 'item bank' OR 'cross-cultural equivalence' OR calibration*):ab,ti,kw) NOT [conference abstract]/lim NOT ([animals]/lim NOT [humans]/lim) AND [english]/lim

Medline ALL Ovid

((((lymphedem* OR lymphoedem* OR oedema OR edema OR swelling*) ADJ6 (size OR measurement* OR volume* OR quanti* OR assess* OR staging OR circumferen*))).ab,ti.) AND (instrumentation.xs. OR methods.xs. OR exp "Validation Studies"/ OR exp "Comparative Study"/ OR exp "psychometrics"/ OR "outcome assessment (health care)"/ OR exp "observer variation"/ OR exp "Health Status Indicators"/ OR exp "reproducibility of results"/ OR exp "discriminant analysis"/ OR (psychometr* OR clinimetr* OR clinometr* OR (outcome ADJ3 (assessment* OR measure*)) OR (observer* ADJ3 variation*) OR reproducib* OR reliab* OR unreliab* OR valid* OR coefficient OR homogeneity OR homogeneous OR (internal* ADJ3 consisten*) OR (cronbach* ADJ3 (alpha OR alphas)) OR (item ADJ3 (correlation* OR selection* OR reduction*)) OR agreement OR precision OR imprecision OR (precise* ADJ3 value*) OR (test ADJ3 retest) OR (reliab* ADJ3 (test OR retest)) OR stabilit* OR interrater* OR inter-rater* OR intrarater* OR intra-rater* OR intertester* OR inter-tester* OR intratester* OR intra-tester* OR interobserver* OR inter-observer* OR intraobserver* OR intra-observer* OR intertechnician* OR inter-technician* OR intratechnician* OR intra-technician* OR interexaminer* OR inter-examiner* OR intraexaminer* OR intra-examiner* OR interassay* OR inter-assay* OR intraassay* OR intra-assay* OR interindividual* OR inter-individual* OR intraindividual* OR intra-individual* OR interparticipant* OR inter-participant* OR intraparticipant* OR intra-participant* OR kappa OR kappa-s OR kappas OR repeatab* OR ((replicab* OR repeated) ADJ3 (measure OR measures OR findings OR result OR results OR test OR tests)) OR generaliza* OR generalisa* OR concordance OR (intraclass ADJ3 correlation*) OR discriminative OR known group OR (factor ADJ (analys* OR structure*)) OR dimension* OR

subscale* OR (multitrait ADJ3 scaling ADJ3 (analysis OR analyses)) OR item discriminant OR (interscale ADJ3 correlation*) OR error OR errors OR ((individual OR interval OR rate OR analysis OR values) ADJ3 variabil*) OR (uncertainty ADJ3 (measurement OR measuring)) OR standard error of measurement OR sensitiv* OR responsive* OR (limit ADJ3 detection) OR minimal detectable concentration OR interpretab* OR ((minimal OR minimally OR clinical OR clinically) ADJ3 (important OR significant OR detectable) ADJ3 (change OR difference)) OR (small* AND (real OR detectable) AND (change OR difference)) OR meaningful change OR ceiling effect OR floor effect OR Item response model OR IRT OR Rasch OR Differential item functioning OR DIF OR computer adaptive testing OR item bank OR cross-cultural equivalence).ab,ti.) NOT (letter OR news OR comment OR editorial OR congresses OR abstracts).pt. NOT ((delphi-technique OR cross-sectional).ti. OR case report/) NOT (exp animals/ NOT humans/) AND english.la.

Web of Science (Science Citation Index Expanded & Social Sciences Citation Index)

((TI=(((lymphedem* OR lymphoedem* OR oedema OR edema) NEAR/5 (size OR measurement* OR volume* OR quanti* OR assess* OR staging OR circumferen*))) OR AB=(((lymphedem* OR lymphoedem* OR oedema OR edema) NEAR/5 (size OR measurement* OR volume* OR quanti* OR assess* OR staging OR circumferen*)))) AND (TI=(psychometr* OR clinimetr* OR clinometr* OR (outcome NEAR/2 (assessment* OR measure*)) OR (observer* NEAR/2 variation*) OR reproducib* OR reliab* OR unreliab* OR valid* OR coefficient OR homogeneity OR homogeneous OR (internal* NEAR/2 consisten*) OR (cronbach* NEAR/2 (alpha OR alphas)) OR (item NEAR/2 (correlation* OR selection* OR reduction*)) OR agreement OR precision OR imprecision OR (precise* NEAR/2 value*) OR (test NEAR/2 retest) OR (reliab* NEAR/2 (test OR retest)) OR stabilit* OR interrater* OR inter-rater* OR intrarater* OR intra-rater* OR intertester* OR inter-tester* OR intratester* OR intra-tester* OR interobserver* OR inter-observer* OR intraobserver* OR intra-observer* OR intertechnician* OR inter-technician* OR intratechnician* OR intra-technician* OR interexaminer* OR inter-examiner* OR intraexaminer* OR intra-examiner* OR interassay* OR inter-assay* OR intraassay* OR intra-assay* OR interindividual* OR inter-individual* OR intraindividual* OR intra-individual* OR interparticipant* OR inter-participant* OR intraparticipant* OR intra-participant* OR kappa OR kappa-s OR kappas OR repeatab* OR ((replicab* OR repeated) NEAR/2 (measure OR measures OR findings OR result OR results OR test OR tests)) OR generaliza* OR generalisa* OR concordance OR (intraclass NEAR/2 correlation*) OR discriminative OR "known group" OR (factor NEAR/1 (analys* OR structure*))

OR dimension* OR subscale* OR (multitrait NEAR/2 scaling NEAR/2 (analysis OR analyses)) OR "item discriminant" OR (interscale NEAR/2 correlation*) OR error OR errors OR ((individual OR interval OR rate OR analysis OR values) NEAR/2 variabil*) OR (uncertainty NEAR/2 (measurement OR measuring)) OR "standard error of measurement" OR sensitiv* OR responsive* OR (limit NEAR/2 detection) OR "minimal detectable concentration" OR interpretab* OR ((minimal OR minimally OR clinical OR clinically) NEAR/2 (important OR significant OR detectable) NEAR/2 (change OR difference)) OR (small* NEAR/2 (real OR detectable) NEAR/2 (change OR difference)) OR "meaningful change" OR " ceiling effect" OR "floor effect" OR "Item response model" OR IRT OR Rasch OR "Differential item functioning" OR DIF OR "computer adaptive testing" OR "item bank" OR "cross-cultural equivalence" OR calibration*) OR AB=(psychometr* OR clinimetr* OR clinometr* OR (outcome NEAR/2 (assessment* OR measure*)) OR (observer* NEAR/2 variation*) OR reproducib* OR reliab* OR unreliab* OR valid* OR coefficient OR homogeneity OR homogeneous OR (internal* NEAR/2 consisten*) OR (cronbach* NEAR/2 (alpha OR alphas)) OR (item NEAR/2 (correlation* OR selection* OR reduction*)) OR agreement OR precision OR imprecision OR (precise* NEAR/2 value*) OR (test NEAR/2 retest) OR (reliab* NEAR/2 (test OR retest)) OR stabilit* OR interrater* OR inter-rater* OR intrarater* OR intra-rater* OR intertester* OR inter-tester* OR intratester* OR intra-tester* OR interobserver* OR inter-observer* OR intraobserver* OR intra-observer* OR intertechnician* OR inter-technician* OR intratechnician* OR intra-technician* OR interexaminer* OR inter-examiner* OR intraexaminer* OR intra-examiner* OR interassay* OR inter-assay* OR intraassay* OR intra-assay* OR interindividual* OR inter-individual* OR intraindividual* OR intra-individual* OR interparticipant* OR inter-participant* OR intraparticipant* OR intra-participant* OR kappa OR kappa-s OR kappas OR repeatab* OR ((replicab* OR repeated) NEAR/2 (measure OR measures OR findings OR result OR results OR test OR tests)) OR generaliza* OR generalisa* OR concordance OR (intraclass NEAR/2 correlation*) OR discriminative OR "known group" OR (factor NEAR/1 (analys* OR structure*)) OR dimension* OR subscale* OR (multitrait NEAR/2 scaling NEAR/2 (analysis OR analyses)) OR "item discriminant" OR (interscale NEAR/2 correlation*) OR error OR errors OR ((individual OR interval OR rate OR analysis OR values) NEAR/2 variabil*) OR (uncertainty NEAR/2 (measurement OR measuring)) OR "standard error of measurement" OR sensitiv* OR responsive* OR (limit NEAR/2 detection) OR "minimal detectable concentration" OR interpretab* OR ((minimal OR minimally OR clinical OR clinically) NEAR/2 (important OR significant OR detectable) NEAR/2 (change OR difference)) OR (small* NEAR/2 (real OR detectable) NEAR/2 (change OR difference)) OR "meaningful change" OR " ceiling effect" OR

"floor effect" OR "Item response model" OR IRT OR Rasch OR "Differential item functioning" OR DIF OR "computer adaptive testing" OR "item bank" OR "cross-cultural equivalence" OR calibration*))) AND DT=(article) AND LA=(english)

CINAHL EBSCOhost

(TI(((lymphedem* OR lymphoedem* OR oedema OR edema OR swelling*) N5 (size OR measurement* OR volume* OR quanti* OR assess* OR staging OR circumferen*))) OR AB (((lymphedem* OR lymphoedem* OR oedema OR edema OR swelling*) N5 (size OR measurement* OR volume* OR quanti* OR assess* OR staging OR circumferen*)))) AND (MH "Validation Studies+" OR MH "Comparative Studies+" OR MH "psychometrics+" OR MH "outcome assessment" OR MH "Observer Bias+" OR MH "Health Status Indicators+" OR MH "reproducibility of results+" OR MH "discriminant analysis+" OR TI(psychometr* OR clinimetr* OR clinometr* OR (outcome N2 (assessment* OR measure*)) OR (observer* N2 variation*) OR reproducib* OR reliab* OR unreliab* OR valid* OR coefficient OR homogeneity OR homogeneous OR (internal* N2 consisten*) OR (cronbach* N2 (alpha OR alphas)) OR (item N2 (correlation* OR selection* OR reduction*)) OR agreement OR precision OR imprecision OR (precise* N2 value*) OR (test N2 retest) OR (reliab* N2 (test OR retest)) OR stabilit* OR interrater* OR inter-rater* OR intrarater* OR intra-rater* OR intertester* OR inter-tester* OR intratester* OR intra-tester* OR interobserver* OR inter-observer* OR intraobserver* OR intra-observer* OR intertechnician* OR inter-technician* OR intratechnician* OR intra-technician* OR interexaminer* OR inter-examiner* OR intraexaminer* OR intra-examiner* OR interassay* OR inter-assay* OR intraassay* OR intra-assay* OR interindividual* OR inter-individual* OR intraindividual* OR intra-individual* OR interparticipant* OR inter-participant* OR intraparticipant* OR intra-participant* OR kappa OR kappa-s OR kappas OR repeatab* OR ((replicab* OR repeated) N2 (measure OR measures OR findings OR result OR results OR test OR tests)) OR generaliza* OR generalisa* OR concordance OR (intraclass N2 correlation*) OR discriminative OR known group OR (factor N1 (analys* OR structure*)) OR dimension* OR subscale* OR (multitrait N2 scaling N2 (analysis OR analyses)) OR item discriminant OR (interscale N2 correlation*) OR error OR errors OR ((individual OR interval OR rate OR analysis OR values) N2 variabil*) OR (uncertainty N2 (measurement OR measuring)) OR standard error of measurement OR sensitiv* OR responsive* OR (limit N2 detection) OR minimal detectable concentration OR interpretab* OR ((minimal OR minimally OR clinical OR clinically) N2 (important OR significant OR detectable) N2 (change OR difference)) OR (small* AND (real OR detectable) AND (change OR difference)) OR meaningful change OR ceiling effect OR floor

effect OR Item response model OR IRT OR Rasch OR Differential item functioning OR DIF OR computer adaptive testing OR item bank OR cross-cultural equivalence) OR AB(psychometr* OR clinimetr* OR clinometr* OR (outcome N2 (assessment* OR measure*)) OR (observer* N2 variation*) OR reproducib* OR reliab* OR unreliab* OR valid* OR coefficient OR homogeneity OR homogeneous OR (internal* N2 consisten*) OR (cronbach* N2 (alpha OR alphas)) OR (item N2 (correlation* OR selection* OR reduction*)) OR agreement OR precision OR imprecision OR (precise* N2 value*) OR (test N2 retest) OR (reliab* N2 (test OR retest)) OR stabilit* OR interrater* OR inter-rater* OR intrarater* OR intra-rater* OR intertester* OR inter-tester* OR intratester* OR intra-tester* OR interobserver* OR inter-observer* OR intraobserver* OR intra-observer* OR intertechnician* OR inter-technician* OR intratechnician* OR intra-technician* OR interexaminer* OR inter-examiner* OR intraexaminer* OR intra-examiner* OR interassay* OR inter-assay* OR intraassay* OR intra-assay* OR interindividual* OR inter-individual* OR intraindividual* OR intra-individual* OR interparticipant* OR inter-participant* OR intraparticipant* OR intra-participant* OR kappa OR kappa-s OR kappas OR repeatab* OR ((replicab* OR repeated) N2 (measure OR measures OR findings OR result OR results OR test OR tests)) OR generaliza* OR generalisa* OR concordance OR (intraclass N2 correlation*) OR discriminative OR known group OR (factor N1 (analys* OR structure*)) OR dimension* OR subscale* OR (multitrait N2 scaling N2 (analysis OR analyses)) OR item discriminant OR (interscale N2 correlation*) OR error OR errors OR ((individual OR interval OR rate OR analysis OR values) N2 variabil*) OR (uncertainty N2 (measurement OR measuring)) OR standard error of measurement OR sensitiv* OR responsive* OR (limit N2 detection) OR minimal detectable concentration OR interpretab* OR ((minimal OR minimally OR clinical OR clinically) N2 (important OR significant OR detectable) N2 (change OR difference)) OR (small* AND (real OR detectable) AND (change OR difference)) OR meaningful change OR ceiling effect OR floor effect OR Item response model OR IRT OR Rasch OR Differential item functioning OR DIF OR computer adaptive testing OR item bank OR cross-cultural equivalence)) NOT PT(letter OR news OR comment OR editorial OR congresses OR abstracts) NOT (TI(delphi-technique OR cross-sectional) OR MH case report) NOT (MH animals+ NOT MH humans) AND LA(english)
