# Supplementary material for: The diagnostic accuracy of soft tissue oedema measurements: a systematic review and best-evidence synthesis
Source: Support Care Cancer. 2026 Mar 5;34(3):281. doi: 10.1007/s00520-026-10373-y (PMC12960331; doi:10.1007/s00520-026-10373-y)
Supplement: Supplementary file 2 — Supplementary Material 2 (PDF 113 KB) [file 520_2026_10373_MOESM2_ESM.pdf]

Margje B. Buitenhuis, Elise M. Gane, Janine T. Hidding, Judith D. de Rooij, Wichor M. Bramer, Remco de Bree, Caroline M. Speksnijder, **The diagnostic accuracy of soft tissue oedema measurements: a systematic review and best-evidence synthesis**, *Support Care Cancer*
Corresponding author: Caroline M. Speksnijder, Department of Oral and Maxillofacial Surgery and Department of Head and Neck Surgical Oncology, University Medical Center Utrecht, Utrecht University, Utrecht, The Netherlands, e-mail: C.M.Speksnijder@umcutrecht.nl

Supplementary information 2. Methodological assessment guide

Table 1. Assessment guide for methodological quality assessment according to the Quality Assessment of Diagnostic Accuracy Studies tool (QUADAS-2). Scores +, ?, and – mean low, unclear, and high risk of bias, respectively.

Item	+	?	-
**1. Patient selection**			
**a. Enrollment**	Consecutive or random, explicitly stated in article	Information not available or unclear from article	Not consecutive or random
**b. Design**	No case control	Information not available or unclear from article	Case control design
**c. Exclusions**	Inappropriate exclusions were avoided. Information should be given about eligibility criteria.	Information not available or unclear from article	Inappropriate exclusions
**d. Overall**	All items (a, b and c) classified as ‘+’	One or more items (a, b or c) classified as ‘?’ and no items classified as ‘-’	One or more items (a, b or c) classified as ‘-’
**e. Applicability**	Patients match the review question	Information not available or unclear from article	Concerns that patients does not match the review question
**2. Index measurement instrument**			
**a. Blinding**	Index test results were interpreted without knowledge of the results of the reference standard	Information not available or unclear from article	Index test results were interpreted with knowledge of the reference standard, or index test and reference standard are different outcome measures of the same measurement instrument
**b. Threshold**	Threshold was pre-specified. The index test should be clearly described as well how the diagnosis was determined	Information not available or unclear from article	Threshold used was determined after the test was applied
**c. Overall**	All items (a and b) classified as ‘+’	One or more items (a or b) classified as ‘?’ and no items classified as ‘-’	One or more items (a or b) classified as ‘-’

**d. Applicability**	Index measurement instrument match the review question	Information not available or unclear from article	Concerns that index measurement instrument does not match the review question
**3. Reference measurement instrument**			
**a. Diagnosis**	the reference standard is likely to correctly classify the target condition and states the threshold for diagnosis	Information not available or unclear from article, or the article does not state the threshold for diagnosis	The reference standard is not likely to correctly classify the target condition
**b. Blinding**	The reference standard results were interpreted without knowledge of the results of the index test	Information not available or unclear from article	The reference standard results were interpreted with knowledge of the results of the index test, or index test and reference standard are different outcome measures of the same measurement instrument
**c. Overall**	All items (a and b) classified as '+'	One or more items (a or b) classified as '?' and no items classified as '-'	One or more items (a or b) classified as '-'
**d. Applicability**	Reference measurement instrument match the review question	Information not available or unclear from article	Concerns that reference measurement instrument does not match the review question
**4. Flow and timing**			
**a. Interval**	Maximal 1 day between the index test and reference standard	Information not available or unclear from article	More than 1 day between the index test and reference standard
**b. Reference**	All patients received the same reference standard	Information not available or unclear from article	Not all patients received the same reference standard
**c. Exclusions**	All patients were included in the analysis without any drop outs	Information not available or unclear from article	Not all patients were included in the analysis
**d. Overall**	All items (a, b and c) classified as '+'	One or more items (a, b or c) classified as '?' and no items classified as '-'	One or more items (a, b or c) classified as '-'

Table 2 Assessment guide for methodological quality assessment according to the COnsensus-based Standards for the selection of health Measurement Instruments (COSMIN) checklist Box 8.

	Very good	Doubtful	Inadequate	NA

**Statistical methods**	Sensitivity and specificity calculated		Sensitivity and specificity not calculated	Not applicable
**Other**	No other important methodological flaws	Other minor methodological flaws, e.g.<br>• Same patients multiple times in analysis<br>• Significant difference between healthy and LE groups (in case of case-control)<br>• Groups are not compared for gender and age (in case of case-control)<br>• No information given about thresholds of reference instrument or index test<br>• Reference instrument is baseline measurement of index test.	Other important methodological flaws<br><br>e.g.<br>• Same measurement instrument for index test and reference<br>• No information given about how construct of reference instrument or index test is obtained	
