# Supplementary material for: The diagnostic accuracy of soft tissue oedema measurements: a systematic review and best-evidence synthesis
Source: Support Care Cancer. 2026 Mar 5;34(3):281. doi: 10.1007/s00520-026-10373-y (PMC12960331; doi:10.1007/s00520-026-10373-y)
Supplement: Supplementary file 3 — Supplementary Material 3 (PDF 30.2 KB) [file 520_2026_10373_MOESM3_ESM.pdf]

Margje B. Buitenhuis, Elise M. Gane, Janine T. Hidding, Judith D. de Rooij, Wichor M. Bramer, Remco de Bree, Caroline M. Speksnijder, **The diagnostic accuracy of soft tissue oedema measurements: a systematic review and best-evidence synthesis**, *Support Care Cancer.*
Corresponding author: Caroline M. Speksnijder, Department of Oral and Maxillofacial Surgery and Department of Head and Neck Surgical Oncology, University Medical Center Utrecht, Utrecht University, Utrecht, The Netherlands, e-mail: C.M.Speksnijder@umcutrecht.nl

Supplementary information 3. Complete quality assessment

Table 1. Complete methodological quality assessment with QUADAS-2 and COSMIN Box 8.

	QUADAS-2																	COSMIN box 8	
**Author, year**	**Patient selection**					**Index measurement instrument**				**Reference measurement instrument**				**Flow and timing**					
	**Enrollment**	**Design**	**Exclusions**	**Overall**	**Applicability**	**Blinding**	**Threshold**	**Overall**	**Applicability**	**Diagnosis**	**Blinding**	**Overall**	**Applicability**	**Interval**	**Reference**	**Exclusions**	**Overall**	**Statistical methods**	**Other**
Aldrich *et al.* [1], 2022	?	+	+	?	+	?	+	?	+	+	?	?	+	+	+	−	−	+	?
Asim *et al.* [2], 2012	+	+	+	+	+	−	−	−	+	+	−	−	+	+	+	−	−	+	−
Bakar *et al.* [3], 2018	?	+	+	?	+	?	+	?	+	?	+	?	+	?	+	+	?	+	+
Barrio *et al.* [4], 2015	?	+	?	?	+	?	+	?	+	+	?	?	+	+	+	+	+	+	?
Berlit *et al.* [5], 2012	?	+	?	?	+	?	−	−	+	?	?	?	+	?	+	+	?	+	?
Berlit *et al.* [6], 2013	?	+	?	?	+	?	−	−	+	?	?	?	+	?	+	−	−	+	?
Bland *et al.* [7], 2003	?	+	+	?	+	?	+	?	+	?	?	?	+	−	−	−	−	+	−
Brandini Da Silva Tozzo *et al.* [8], 2023	−	+	+	−	+	?	+	?	+	+	?	?	+	+	+	−	−	+	+

Author, year	QUADAS-2																	COSMIN box 8	
	Patient selection					Index measurement instrument				Reference measurement instrument				Flow and timing					
	Enrollment	Design	Exclusions	Overall	Applicability	Blinding	Threshold	Overall	Applicability	Diagnosis	Blinding	Overall	Applicability	Interval	Reference	Exclusions	Overall	Statistical methods	Other
Bundred *et al.* [9], 2015	?	+	?	?	+	?	+	?	+	+	?	?	+	?	+	−	−	+	?
Chan *et al.* [10], 2018	+	+	+	+	+	−	−	−	+	+	+	+	+	−	+	−	−	+	+
Chung *et al.* [11], 2006	+	+	+	+	+	+	−	−	+	+	+	+	+	+	+	+	+	+	+
Cornish *et al.* [12], 2001	+	−	+	−	+	?	−	−	+	+	?	?	+	?	+	+	?	+	?
Devoogdt *et al.* [13], 2014	?	+	+	?	+	?	+	?	+	+	+	+	+	?	+	−	−	+	+
Dylke *et al.* [14], 2016	−	−	+	−	+	?	+/−[a]	−	+	+	+	+	+	+	+	−	−	+	?
Dylke *et al.* [15], 2018	?	+	?	?	+	+	−	−	+	?	?	?	?	?	?	−	−	+	−
Erdinç Gündüz *et al.* [16], 2021	?	+	+	?	+	+	−	−	+	+	+	+	+	+	+	+	+	+	?
Erdogan Iyigun *et al.* [17], 2019	?	+	+	?	+	+	−	−	+	+	+	+	+	?	+	+	?	+	+
Fu *et al.* [18], 2013	−	+	+	−	+	?	+/−[a]	−	+	+	+	+	+	?	?	+	?	+	?
Furlan *et al.* [19], 2021	?	+	+	?	+	?	+	?	+	+	?	?	+	?	+	−	−	+	+

Author, year	QUADAS-2																	COSMIN box 8	
	**Patient selection**					**Index measurement instrument**				**Reference measurement instrument**				**Flow and timing**					
	**Enrollment**	**Design**	**Exclusions**	**Overall**	**Applicability**	**Blinding**	**Threshold**	**Overall**	**Applicability**	**Diagnosis**	**Blinding**	**Overall**	**Applicability**	**Interval**	**Reference**	**Exclusions**	**Overall**	**Statistical methods**	**Other**
Giray and Yağcı [20], 2019	?	+	+	?	+	?	−	−	+	+	?	?	+	?	+	+	?	+	+
Godoy *et al.* [21], 2007	+	+	+	+	+	?	+	?	+	+	?	?	+	?	+	+	?	+	+
Hara and Mihara [22], 2021	?	+	?	?	+	+	+	+	+	+	?	?	+	?	+	+	?	+	+
Hayes *et al.* [23], 2005	+	+	+	+	+	?	+	?	+	+	?	?	+	?	+	−	−	+	+
Hayes *et al.* [24], 2008	+	+	+	+	+	?	+	?	+	+	?	?	+	?	+	−	−	+	?
Hidding *et al.* [25], 2018	?	+	+	?	+	−	−	−	+	+	−	−	+	+	+	−	−	+	−
Jeffs and Purushotham [26], 2016	?	+	−	−	+	?	+	?	+	+	?	?	+	+	+	+	+	+	+
Keo *et al.* [27], 2015	?	+	?	?	+	?	−	−	+	+	+	+	+	?	+	+	?	+	+
Keo *et al.* [28], 2013	?	+	?	?	+	?	−	−	+	+	+	+	+	?	+	+	?	+	+
Koo *et al.* [29], 2019	−	+	−	−	+	?	−	−	+	+	?	?	+	?	+	+	?	+	+

Author, year	QUADAS-2																	COSMIN box 8	
	Patient selection					Index measurement instrument				Reference measurement instrument				Flow and timing					
	Enrollment	Design	Exclusions	Overall	Applicability	Blinding	Threshold	Overall	Applicability	Diagnosis	Blinding	Overall	Applicability	Interval	Reference	Exclusions	Overall	Statistical methods	Other
Lahtinen *et al.* [30], 2015	?	+	?	?	+	?	+	?	+	+	?	?	+	?	+	+	?	+	?
Lee *et al.* [31], 2020	?	+	?	?	+	+	−	−	+	+	+	+	+	?	+	+	?	+	+
Li *et al.* [32], 2015	?	+	+	?	+	+	−	−	+	+	+	+	+	+	+	+	+	+	+
Lim *et al.* [33], 2019	?	+	+	?	+	?	−	−	+	+	?	?	+	?	+	+	?	+	+
Liu *et al.* [34], 2022	?	+	+	?	+	?	+	?	+	+	?	?	+	?	+	+	?	−	+
Lopez Penha *et al.* [35], 2011	?	+	+	?	+	?	+	?	+	+	?	?	+	+	+	+	+	+	+
Lu *et al.* [36], 2014	+	+	+	+	+	+	−	−	+	+	+	+	+	?	+	+	?	+	+
Omura *et al.*[37], 2022	?	−	?	−	+	?	−	−	+	+	?	?	+	−	+	?	−	+	+
Pichonnaz *et al.* [38], 2015	+	+	+	+	+	−	−	−	+	+	+	+	+	−	+	−	−	+	?
Riches *et al.* [39], 2023	−	+	+	−	+	?	−	−	+	+	+	+	+	+	+	+	+	+	+
Sampathirao *et al.* [40], 2021	?	−	+	−	+	?	−	−	+	+	+	+	+	?	+	+	?	+	+

Author, year	QUADAS-2																	COSMIN box 8	
	Patient selection					Index measurement instrument				Reference measurement instrument				Flow and timing					
	Enrollment	Design	Exclusions	Overall	Applicability	Blinding	Threshold	Overall	Applicability	Diagnosis	Blinding	Overall	Applicability	Interval	Reference	Exclusions	Overall	Statistical methods	Other
Svensson *et al.* [41], 2020	?	+	+	?	+	?	+	?	+	+	?	?	+	+	+	+	+	+	+
Thomis *et al.* [42], 2020	+	+	+	+	+	+	+	+	+	+	+	+	+	−	+	+	−	+	+
Thomis *et al.* [43], 2022	+	+	+	+	+	−	+	−	+	+	?	?	+	+	+	+	+	+	?
Wang *et al.* [44], 2018	?	+	+	?	+	+	−	−	+	+	+	+	+	+	+	+	+	+	+
Wiser *et al.* [45], 2020	+	+	?	?	+	?	+ / − [a]	−	+	+	?	?	+	?	+	−	−	+	+

[a] Thresholds are prespecified and not prespecified

References

1. Aldrich MB, Rasmussen JC, DeSnyder SM, Woodward WA, Chan W, Sevick-Muraca EM, et al. Prediction of breast cancer-related lymphedema by dermal backflow detected with near-infrared fluorescence lymphatic imaging. Breast Cancer Res Treat. 2022;195(1):33-41.
2. Asim M, Cham A, Banerjee S, Nancekivell R, Dutu G, McBride C, et al. Difficulties with defining lymphoedema after axillary dissection for breast cancer. N Z Med J. 2012;125(1351):29-39.
3. Bakar Y, Tugral A, Uyeturk U. Measurement of Local Tissue Water in Patients with Breast Cancer-Related Lymphedema. Lymphat Res Biol. 2018;16(2):160-4.
4. Barrio AV, Eaton A, Frazier TG. A Prospective Validation Study of Bioimpedance with Volume Displacement in Early-Stage Breast Cancer Patients at Risk for Lymphedema. Ann Surg Oncol. 2015;22 Suppl 3(0 3):S370-5.
5. Berlit S, Brade J, Tuschy B, Hornemann A, Leweling H, Eghardt V, et al. Comparing bioelectrical impedance values in assessing early upper limb lymphedema after breast cancer surgery. In Vivo. 2012;26(5):863-7.
6. Berlit S, Brade J, Tuschy B, Hornemann A, Leweling H, Eghardt V, et al. Whole-body Bioelectrical Impedance Analysis in Assessing Upper-limb Lymphedema After Breast Cancer Therapy. Anticancer Research. 2013;33(10):4553-6.
7. Bland KL, Perczyk R, Du W, Rymal C, Koppolu P, McCrary R, et al. Can a practicing surgeon detect early lymphedema reliably? Am J Surg. 2003;186(5):509-13.
8. Brandini da Silva Tozzo FC, Sarri AJ, Pirola WE, Cardoso da Silva UB, de Oliveira MA, de Pádua Souza C, et al. Evaluation of upper limb lymphoedema and diagnostic accuracy of bioimpedance spectroscopy. A comprehensive validation in a Brazilian population. ecancermedicalscience. 2023;17:1649.
9. Bundred NJ, Stockton C, Keeley V, Riches K, Ashcroft L, Evans A, et al. Comparison of multi-frequency bioimpedance with perometry for the early detection and intervention of lymphoedema after axillary node clearance for breast cancer. Breast Cancer Research and Treatment. 2015;151(1):121-9.
10. Chan WH, Huang YL, Lin C, Lin CY, Cheng MH, Chu SY. Acoustic Radiation Force Impulse Elastography: Tissue Stiffness Measurement in Limb Lymphedema. Radiology. 2018;289(3):759-65.
11. Chung YH, Chao TY, Chiu CT, Lin MC. The cuff-leak test is a simple tool to verify severe laryngeal edema in patients undergoing long-term mechanical ventilation. Critical Care Medicine. 2006;34(2):409-14.
12. Cornish BH, Chapman M, Hirst C, Mirolo B, Bunce IH, Ward LC, et al. Early diagnosis of lymphedema using multiple frequency bioimpedance. Lymphology. 2001;34(1):2-11.
13. Devoogdt N, Pans S, De Groef A, Geraerts I, Christiaens MR, Neven P, et al. Postoperative Evolution of Thickness and Echogenicity of Cutis and Subcutis of Patients With and Without Breast Cancer-Related Lymphedema. Lymphatic Research and Biology. 2014;12(1):23-31.
14. Dylke ES, Schembri GP, Bailey DL, Bailey E, Ward LC, Refshauge K, et al. Diagnosis of upper limb lymphedema: development of an evidence-based approach. Acta Oncol. 2016;55(12):1477-83.
15. Dylke ES, Benincasa Nakagawa H, Lin L, Clarke JL, Kilbreath SL. Reliability and Diagnostic Thresholds for Ultrasound Measurements of Dermal Thickness in Breast Lymphedema. Lymphat Res Biol. 2018;16(3):258-62.
16. Erdinç Gündüz N, Dilek B, Şahin E, Ellidokuz H, Akalın E. Diagnostic Contribution of Ultrasonography in Breast Cancer-Related Lymphedema. Lymphatic Research and Biology. 2021;19(6):517-23.
17. Erdogan Iyigun Z, Agacayak F, Ilgun AS, Elbuken Celebi F, Ordu C, Alco G, et al. The Role of Elastography in Diagnosis and Staging of Breast Cancer-Related Lymphedema. Lymphat Res Biol. 2019;17(3):334-9.
18. Fu MR, Cleland CM, Guth AA, Kayal M, Haber J, Cartwright F, et al. L-dex ratio in detecting breast cancer-related lymphedema: reliability, sensitivity, and specificity. Lymphology. 2013;46(2):85-96.
19. Furlan C, Matheus CN, Jales RM, Derchain SFM, Bennini JR, Jr., Sarian LO. Longitudinal, Long-Term Comparison of Single- versus Multipoint Upper Limb Circumference Periodical Measurements as a Tool to Predict Persistent Lymphedema in Women Treated Surgically for Breast Cancer: An Optimized Strategy to Early Diagnose Lymphedema and Avoid Permanent Sequelae in Breast Cancer Survivors. Ann Surg Oncol. 2021;28(13):8665-76.
20. Giray E, Yagci I. Diagnostic accuracy of interlimb differences of ultrasonographic subcutaneous tissue thickness measurements in breast cancer-related arm lymphedema. Lymphology. 2019;52(1):1-10.
21. Godoy JM, Silva SH, Godoy MF. Sensitivity and specificity of combined perimetric and volumetric evaluations in the diagnosis of arm lymphedema. Prague Med Rep. 2007;108(3):243-7.
22. Hara H, Mihara M. Diagnosis of Lymphatic Dysfunction by Evaluation of Lymphatic Degeneration with Lymphatic Ultrasound. Lymphat Res Biol. 2021;19(4):334-9.
23. Hayes S, Cornish B, Newman B. Comparison of methods to diagnose lymphoedema among breast cancer survivors: 6-month follow-up. Breast Cancer Res Treat. 2005;89(3):221-6.
24. Hayes S, Janda M, Cornish B, Battistutta D, Newman B. Lymphedema secondary to breast cancer: how choice of measure influences diagnosis, prevalence, and identifiable risk factors. Lymphology. 2008;41(1):18-28.
25. Hidding JT, Beurskens CHG, De Vries MT, Nijhuis-van der Sanden MWG, van Laarhoven HWM, van der Wees PJ. Accuracy of a single measurement site for self-monitoring of patients with breast cancer at risk for lymphedema. Physiother Theory Pract. 2019;35(12):1322-7.
26. Jeffs E, Purushotham A. The prevalence of lymphoedema in women who attended an information and exercise class to reduce the risk of breast cancer-related upper limb lymphoedema. Springerplus. 2016;5:21.
27. Keo HH, Husmann M, Groechenig E, Willenberg T, Gretener SB. Diagnostic accuracy of fluorescence microlymphography for detecting limb lymphedema. Eur J Vasc Endovasc Surg. 2015;49(4):474-9.

28. Keo HH, Schilling M, Buchel R, Grochenig E, Engelberger RP, Willenberg T, et al. Sensitivity and specificity of fluorescence microlymphography for detecting lymphedema of the lower extremity. Vasc Med. 2013;18(3):117-21.
29. Koo KI, Ko MH, Lee Y, Son HW, Lee S, Ho Hwang C. Comparison of a novel algorithm quantitatively estimating epifascial fibrosis in three-dimensional computed tomography images to other clinical lymphedema grading methods. PLoS ONE. 2019;14(12).
30. Lahtinen T, Seppala J, Viren T, Johansson K. Experimental and Analytical Comparisons of Tissue Dielectric Constant (TDC) and Bioimpedance Spectroscopy (BIS) in Assessment of Early Arm Lymphedema in Breast Cancer Patients after Axillary Surgery and Radiotherapy. Lymphat Res Biol. 2015;13(3):176-85.
31. Lee YL, Huang YL, Chu SY, Chan WH, Cheng MH, Lin YH, et al. Characterization of limb lymphedema using the statistical analysis of ultrasound backscattering. Quantitative Imaging in Medicine and Surgery. 2020;10(1):48-56.
32. Li Y, Lu Q, Chen TW, Yao Y, Zhao Z, Li Y, et al. Thickness of soft tissue of lower extremities measured with magnetic resonance imaging as a new indicator for staging unilateral secondary lower extremity lymphedema. Acta Radiol. 2015;56(8):1016-24.
33. Lim SM, Han Y, Kim SI, Park HS. Utilization of bioelectrical impedance analysis for detection of lymphedema in breast Cancer survivors: a prospective cross sectional study. BMC Cancer. 2019;19(1):669.
34. Liu Y, Long X, Guan J. Tissue Dielectric Constant Combined With Arm Volume Measurement as Complementary Methods in Detection and Assessment of Breast Cancer-Related Lymphedema. Lymphat Res Biol. 2022;20(1):26-32.
35. Lopez Penha TR, Slangen JJ, Heuts EM, Voogd AC, Von Meyenfeldt MF. Prevalence of lymphoedema more than five years after breast cancer treatment. Eur J Surg Oncol. 2011;37(12):1059-63.
36. Lu Q, Li Y, Chen TW, Yao Y, Zhao Z, Li Y, et al. Validity of soft-tissue thickness of calf measured using MRI for assessing unilateral lower extremity lymphoedema secondary to cervical and endometrial cancer treatments. Clin Radiol. 2014;69(12):1287-94.
37. Omura M, Saito W, Akita S, Yoshida K, Yamaguchi T. In Vivo Quantitative Ultrasound on Dermis and Hypodermis for Classifying Lymphedema Severity in Humans. Ultrasound Med Biol. 2022;48(4):646-62.
38. Pichonnaz C, Bassin JP, Lécureux E, Currat D, Jolles BM. Bioimpedance spectroscopy for swelling evaluation following total knee arthroplasty: A validation study. BMC Musculoskelet Disord. 2015:1-8.
39. Riches K, Cheung KL, Keeley V. Improving the Assessment and Diagnosis of Breast Lymphedema after Treatment for Breast Cancer. Cancers (Basel). 2023;15(6):1758.
40. Sampathirao N, Indirani M, Manokaran G, Jaykanth A, Patel A, Simon S. Assessment of lymphedema with lymphoscintigraphy: Can nodal quantification help? Lymphology. 2021;54(2):92-105.
41. Svensson BJ, Dylke ES, Ward LC, Black DA, Kilbreath SL. Screening for breast cancer–related lymphoedema: self-assessment of symptoms and signs. Supportive Care Cancer. 2020;28(7):3073-80.
42. Thomis S, Dams L, Fourneau I, De Vrieze T, Nevelsteen I, Neven P, et al. Correlation Between Clinical Assessment and Lymphofluoroscopy in Patients with Breast Cancer-Related Lymphedema: A Study of Concurrent Validity. Lymphat Res Biol. 2020.
43. Thomis S, Devoogdt N, De Vrieze T, Bechter-Hugl B, Heroes AK, Fourneau I. Relation Between Early Disturbance of lymphatic transport Visualized With Lymphofluoroscopy and Other Clinical Assessment Methods in Patients With Breast Cancer. Clin Breast Cancer. 2022;22(1):e37-e47.
44. Wang L, Wu X, Wu M, Zhao Z, Tang H, Li S, et al. Edema Areas of Calves Measured with Magnetic Resonance Imaging as a Novel Indicator for Early Staging of Lower Extremity Lymphedema. Lymphatic Res Biol. 2018;16(3):240-7.
45. Wiser I, Mehrara BJ, Coriddi M, Kenworthy E, Cavalli M, Encarnacion E, et al. Preoperative assessment of upper extremity secondary lymphedema. Cancers. 2020;12(1).
